# Further delineation of Malan syndrome

**DOI:** 10.1002/humu.23563

**Published:** 2018-06-25

**Authors:** Manuela Priolo, Denny Schanze, Katrin Tatton‐Brown, Paul A. Mulder, Jair Tenorio, Kreepa Kooblall, Inés Hernández Acero, Fowzan S. Alkuraya, Pedro Arias, Laura Bernardini, Emilia K. Bijlsma, Trevor Cole, Christine Coubes, Irene Dapia, Sally Davies, Nataliya Di Donato, Nursel H. Elcioglu, Jill A. Fahrner, Alison Foster, Noelia García González, Ilka Huber, Maria Iascone, Ann‐Sophie Kaiser, Arveen Kamath, Jan Liebelt, Sally Ann Lynch, Saskia M. Maas, Corrado Mammì, Inge B. Mathijssen, Shane McKee, Leonie A. Menke, Ghayda M. Mirzaa, Tara Montgomery, Dorothee Neubauer, Thomas E. Neumann, Letizia Pintomalli, Maria Antonietta Pisanti, Astrid S. Plomp, Sue Price, Claire Salter, Fernando Santos‐Simarro, Pierre Sarda, Mabel Segovia, Charles Shaw‐Smith, Sarah Smithson, Mohnish Suri, Rita Maria Valdez, Arie Van Haeringen, Johanna M. Van Hagen, Marcela Zollino, Pablo Lapunzina, Rajesh V. Thakker, Martin Zenker, Raoul C. Hennekam

**Affiliations:** ^1^ Unità Operativa di Genetica Medica Grande Ospedale Metropolitano Bianchi‐Melacrino‐Morelli Reggio Calabria Italy; ^2^ Institute of Human Genetics University Hospital Magdeburg Magdeburg Germany; ^3^ Division of Genetics and Epidemiology Institute of Cancer Research London and South West Thames Regional Genetics Service St. George's University Hospitals NHS Foundation Trust London UK; ^4^ Autism Team Northern‐Netherlands Jonx Department of Youth Mental Health Lentis Psychiatric Institute Groningen The Netherlands; ^5^ Institute of Medical and Molecular Genetics (INGEMM) Hospital Universitario La Paz IdiPAZ Universidad Autónoma de Madrid and CIBERER Centro de Investigación Biomédica en Red de Enfermedades Raras ISCIII Madrid Spain; ^6^ Academic Endocrine Unit Radcliffe Department of Medicine University of Oxford Oxford UK; ^7^ Genetics Unit, Hospital Universitario Central de Asturias Oviedo Spain; ^8^ Saudi Human Genome Project King Abdulaziz City for Science and Technology and Department of Genetics King Faisal Specialist Hospital and Research Center Riyadh Saudi Arabia; ^9^ Cytogenetics Unit Casa Sollievo della Sofferenza Foundation San Giovanni Rotondo Italy; ^10^ Department of Clinical Genetics Leiden University Medical Centre Leiden The Netherlands; ^11^ Department of Clinical Genetics Birmingham Women's and Children's NHS Foundation Trust Birmingham UK; ^12^ Département de Génétique Médicale Hôpital Arnaud de Villeneuve CHRU Montpellier Montpellier France; ^13^ Institute of Medical Genetics University Hospital of Wales Cardiff UK; ^14^ Institute for Clinical Genetics TU Dresden Dresden Germany; ^15^ Department of Pediatric Genetics Marmara University Medical School, Istanbul, and Eastern Mediterranean University Mersin Turkey; ^16^ McKusick‐Nathans Institute of Genetic Medicine Department of Pediatrics Johns Hopkins University School of Medicine Baltimore Maryland; ^17^ Institute of Cancer and Genomic Sciences, College of Medical and Dental Sciences University of Birmingham Birmingham UK; ^18^ Unit Hospital Universitario Central de Asturias Oviedo Spain; ^19^ Sørland Hospital Kristiansand Norway; ^20^ Laboratorio di Genetica Medica ASST Papa Giovanni XXIII Bergamo Italy; ^21^ Institute of Human Genetics Heidelberg University Heidelberg Germany; ^22^ Institute of Medical Genetics University Hospital of Wales Cardiff UK; ^23^ South Australian Clinical Genetics Services SA Pathology North Adelaide Australia; ^24^ UCD Academic Centre on Rare Diseases School of Medicine and Medical Sciences University College Dublin and Clinical Genetics Temple Street Children's University Hospital Dublin Ireland; ^25^ Department of Clinical Genetics Academic Medical Center Amsterdam The Netherlands; ^26^ Belfast HSC Trust Northern Ireland Regional Genetics Service Belfast Northern Ireland; ^27^ Department of Pediatrics Academic Medical Center University of Amsterdam Amsterdam The Netherlands; ^28^ Center for Integrative Brain Research Seattle Children's Research Institute and Department of Human Genetics, University of Washington Seattle Washington; ^29^ Newcastle upon Tyne NHS Foundation Trust Newcastle upon Tyne UK; ^30^ Mitteldeutscher Praxisverbund Humangenetik Halle Germany; ^31^ Medical Genetic and Laboratory Unit “Antonio Cardarelli” Hospital Naples Italy; ^32^ Department of Clinical Genetics Northampton General Hospital NHS Trust Northampton UK; ^33^ Wessex Clinical Genetics Service Princess Ann Hospital Southampton UK; ^34^ CENAGEM Centro Nacional de Genética Buenos Aires Argentina; ^35^ Royal Devon and Exeter NHS Foundation Trust Exeter UK; ^36^ University Hospitals Bristol NHS Trust Bristol UK; ^37^ Nottingham Clinical Genetics Service Nottingham University Hospitals NHS Trust Nottingham UK; ^38^ Genetics Unit, Hospital Militar Central “Cirujano Mayor Dr. Cosme Argerich,” Buenos Aires Argentina; ^39^ Department of Clinical Genetics VU University Medical Centre Amsterdam The Netherlands; ^40^ Department of Laboratory Medicine Institute of Medical Genetics Catholic University Rome Italy

**Keywords:** Malan syndrome, Marshall‐Smith syndrome, NFIX, phenotype, phenotype‐genotype, Sotos syndrome, Weaver syndrome

## Abstract

Malan syndrome is an overgrowth disorder described in a limited number of individuals. We aim to delineate the entity by studying a large group of affected individuals. We gathered data on 45 affected individuals with a molecularly confirmed diagnosis through an international collaboration and compared data to the 35 previously reported individuals. Results indicate that height is > 2 SDS in infancy and childhood but in only half of affected adults. Cardinal facial characteristics include long, triangular face, macrocephaly, prominent forehead, everted lower lip, and prominent chin. Intellectual disability is universally present, behaviorally anxiety is characteristic. Malan syndrome is caused by deletions or point mutations of *NFIX* clustered mostly in exon 2. There is no genotype‐phenotype correlation except for an increased risk for epilepsy with 19p13.2 microdeletions. Variants arose de novo, except in one family in which mother was mosaic. Variants causing Malan and Marshall‐Smith syndrome can be discerned by differences in the site of stop codon formation. We conclude that Malan syndrome has a well recognizable phenotype that usually can be discerned easily from Marshall–Smith syndrome but rarely there is some overlap. Differentiation from Sotos and Weaver syndrome can be made by clinical evaluation only.

## 1 INTRODUCTION

Overgrowth has been defined as “global or regional excess growth compared either to an equivalent body part or the age‐related peer group” (Tatton‐Brown & Weksberg, 2013). Overgrowth syndromes form a group of genetically determined disorders in which at least height is equal to or greater than two standard deviations (SDS) above the mean, but also weight and head circumference may be increased, and the growth pattern is typically present from birth on. The overgrowth may or may not be associated with malformations or dysplasias.

Malan syndrome (MIM# 614753; also called as Sotos syndrome 2) is an overgrowth disorder, characterized by overgrowth, an unusual facial phenotype, intellectual disability, and behavioral problems. It is caused by heterozygous variants or deletions of the gene nuclear factor I X (*NFIX*; MIM# 164005*)*, located at chromosome 19p13.2 (Malan et al., 2010). Since the first description the entity has been described in 35 individuals, to date (Auvin, Holder‐Espinasse, Lamblin, & Andrieux, 2009; Bonaglia et al., 2010; Dolan et al., 2010; Dong et al., 2016; Gurrieri et al., 2015; Jezela‐Stanek et al., 2016; Jorge et al., 2015; Klaassens et al., 2015; Kuroda et al., 2017; Lu et al., 2017; Lysy et al., 2009; Martinez et al., 2015; Natiq et al., 2014; Nimmakayalu et al., 2013; Oshima et al., 2017; Priolo et al., 2012; Shimojima et al., 2015; Yoneda et al., 2012). Haploinsufficiency of *NFIX* has been proposed as leading causative mechanism in Malan syndrome (Gurrieri et al., 2015; Klaassens et al., 2015; Malan et al., 2010). The syndrome is allelic to Marshall‐Smith syndrome (MIM# 602535) (Martinez et al., 2015; Schanze et al., 2014; Shaw et al., 2010), characterized by a dysostosis, postnatal failure to thrive, short stature, unusual face, respiratory compromise, and moderate to severe developmental delay (Shaw et al., 2010; Van Balkom et al., 2011). *NFIX* variants in Marshall‐Smith syndrome were predicted to lead to abnormal proteins with an abnormal C‐terminus while their DNA binding and dimerization domain was preserved. Some variants escape nonsense‐mediated mRNA decay (NMD), suggesting a dominant‐negative effect of mutant NFIX proteins (Malan et al., 2010; Schanze et al., 2014).

Here, we report on 45 individuals with Malan syndrome (14 being mentioned in a table without clinical data (Tatton‐Brown et al., 2017), the others unpublished), provide detailed clinical descriptions and their genotypes, review earlier reported patients, and compare data to those of Marshall–Smith syndrome and to Sotos syndrome and Weaver syndrome (MIM# 277590). We refine genotype‐phenotype correlations in individuals with disease‐causing *NFIX* variants.

## 2 METHODS

### 2.1 Patients

Patients were referred for clinical (R.C.H., M.P) or molecular (M.P., M.Z.) diagnosis, management advices (R.C.H.), or specifically for this study. Patients were typically recognized by their referring physicians using targeted exome sequencing aimed at detecting causes for intellectual disability. In three patients, a clinical diagnosis of Malan syndrome was molecularly confirmed by targeted Sanger sequencing.

A dedicated questionnaire was used to gather clinical and molecular data, and clinical photographs were available on every patient. The facial characteristics were scored independently by two authors (M.P., R.C.H.). In case of inconsistencies in scoring these were discussed till consensus.

### 2.2 Molecular studies

Sanger sequencing and exome sequencing were performed according to local protocols. Confirmation of exome results by Sanger sequencing was performed in most patients, unless exome results were thought sufficiently reliable. Microdeletions were identified by array CGH or SNP array.

Total RNA was extracted from skin fibroblast cultures and cDNA was generated using the SuperScript™ First‐Strand Synthesis System (Thermo Fisher). Specific oligonucleotide primer combinations were used to amplify cDNA fragments of interest, allowing discrimination between different *NFIX* transcript isoforms (Supporting Information Figure 1). Analysis of these fragments was performed by Sanger sequencing.

### 2.3 In silico analysis of mutant NFIX proteins

Combined Annotation Dependent Depletion (CADD; version 1.3; https://cadd.gs.washington.edu/) scores were used as tool for scoring deleteriousness of all variants (Kircher et al., 2014). We performed in silico analysis of predicted mutant cDNAs after ExPASy translation and alignment with wild‐type protein by Clustal Omega (v1.2.4; https://www.ebi.ac.uk/Tools/msa/clustalo/) for short insertions/deletions located in exons 6, 7, and 8 and in variants associated with Marshall‐Smith syndrome. We performed in silico alignment by Clustal Omega of human NFIX protein isoforms encoded by transcripts reported in Ensembl Genome Browser 90 and alignment of paraloges and orthologes to human NFIX.

### 2.4 Literature search

PubMed was searched for publications using as terms “Malan syndrome” OR “Malan overgrowth” OR “Sotos type 2″ OR “Sotos type II” OR “NFIX overgrowth” OR “deletion 19p13.2″ OR “microdeletion 19p13.2″. Exclusion criterion was publication in a language other than English, French, Dutch, Italian, Spanish, or Portuguese. Reference lists of thus obtained manuscripts were hand searched for further references. We excluded reports describing deletions extending centromerically  > 1 Mb from *NFIX* because of the large number of potentially confounding genes. Facial phenotypes of patients depicted in publications were scored independently by two authors (M.P., R.C.H.). Available descriptions of phenotypes were accepted unless photographic evidence of differences was clear.

### 2.5 Ethics

All participants or their legal guardians gave written informed consent for molecular analyses and publication of clinical and genotype data and facial photographs. The study was approved by the medical ethics committee of Great Metropolitan Hospital Bianchi‐Melacrino‐Morelli in Reggio Calabria (N°200 approval).

## 3 RESULTS

**Figure 1:**
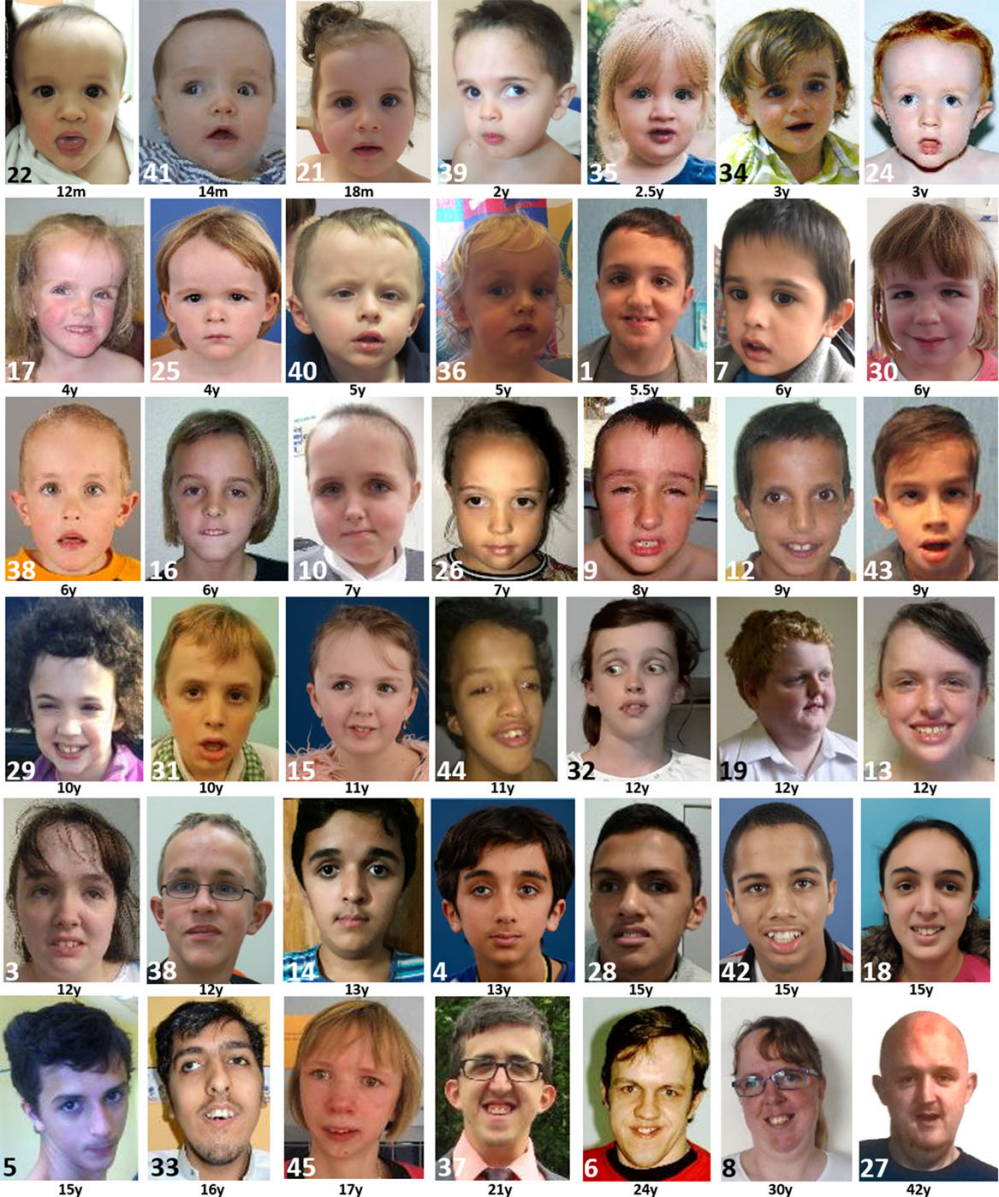
AP facial view of presently reported 42 individuals with Malan syndrome. Numbers of individuals in the panels correspond to numbers in the Tables. Ages are mentioned below each picture. For detailed descriptions please see Tables and text

**Figure 2:**
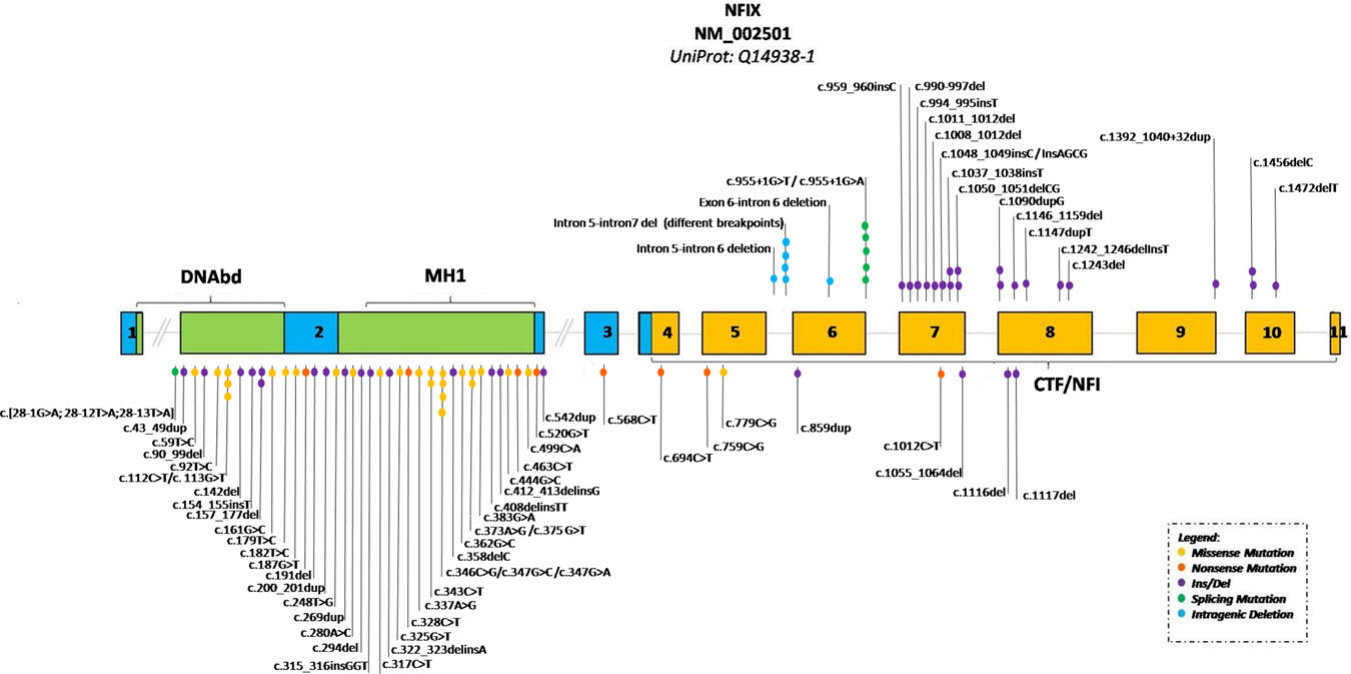
*NFIX* cartoon with all reported variants in Malan (underneath the gene) and Marshall‐Smith (above the gene) syndrome. The color legend for missense, nonsense, ins/dels, splicing, and intragenic deletions is shown. Recurring variants are reported with additional circles. Exons and introns are in scale except introns 1 and 2. Blue: putative DNA binding and dimerization domain of the gene and inside it; green: MH1 (MAD homology 1) domain and the N‐terminal DNA binding (DNAbd) domain; orange: CAAT‐box transcription factor–nuclear factor I (CTF‐NFI) domain

We collected 45 patients with molecularly confirmed Malan syndrome. Three had variably sized microdeletions involving 19p13.2 and 42 had variants in *NFIX*. Literature searches yielded 21 patients with microdeletions involving 19p13.2 and 14 patients with intragenic *NFIX* variants. A summary of main clinical characteristics compared to those in Marshall‐Smith syndrome (Shaw et al., 2010), Sotos syndrome (Tatton‐Brown et al., 2007) and Weaver syndrome (Tatton‐Brown et al., 2013), is provided in Table 1, and illustrated in Figure 1 and Supporting Information Figure 7. Detailed individual patient data are provided in Supporting Materials (Supporting Information Tables 1 and 2). Molecular data are summarized in Figure 2 and Table 2. We discuss here only those results that are not available in the tables.

**Table 1:** Comparison of characteristics of Malan syndrome caused by *NFIX* mutations and by deletions of the complete gene, to those of Marshall‐Smith syndrome, Sotos syndrome, and Weaver syndrome

		Malan syndrome					
		NFIX mutations	NFIX deletions	All	Marshall‐Smith syndrome	Sotos syndrome	Weaver syndrome
**Growth**	Prenatal: Weight at birth < 2SDS	0	0	0	11/55	‐	‐
	Prenatal: Weight at birth > 2SDS	5/54	6/21	11/75	8/55	+++^a^	++
	Postnatal: Height < 2SDS	0	0	0	38/39	‐	‐
	Postnatal: Height > 2SDS	31/55	13/24	44/79	0/18	+++	+++
**Development**	Intellectual disability/global developmental delay	56/56	24/24	80/80	39/39	+++^b^	+++^c^
	Autistic features (A)/anxieties (F)	A 18/52; F 34/54	A 5/22;F 6/21	A 23/74; F 40/75	Na	?	(F) +
**Neurology**	Hypotonia (Ho)/hypertonia (He)	Ho 36/51; He n.a	Ho 21/24; He n.a	Ho 57/75	Ho12/28 He4/28	Ho ++	Ho ++, He ++
	Seizures/EEG anomalies	10/55	11/24	21/79	4/38	++	‐
	Brain anomalies: WV/C/BA/CM	19/44 13WV; 8C; 3CM	11/19 4WV; 6C; 2BA; 3CM	30/63 17WV; 14C; 2BA; 6CM	12/39 C 8; SC 1; WV	WV++, C‐; BA‐; CM‐	+
**Craniofacial morphology**	Macrocephaly	44/55	16/24	60/79	0/39	+++	++
	Long/triangular face	47/55	20/24	67/79	0/57	+++	‐
	Prominent forehead	53/55	24/24	77/79	53/54	+++	+++
	Proptosis	0	1/23	1/78	55/56	‐	‐
	Underdeveloped midface	0	0	0	38/42	‐	‐
	Short nose	26/54	14/21	40/75	43/50	‐	+++
	Anteverted nares	28/54	15/22	43/76	44/53	‐	+++
	Everted lips	40/54	14/21	54/75	13/19	+	‐
	Small chin; prominent chin	1/55; 41/55	1/24; 16/21	2/79; 57/76	55/57	‐	+++
**Eyes**	Vision impaired	42/56	18/24	60/80	9/18	+	+
	Blue sclerae	15/52	4/14	19/66	34/41	‐	‐
**Respiratory tract**	Airway obstructions	1/55	0	1/78	45/55	‐	‐
**Musculo‐skeletal anomalies**	Abnormal bone maturation	29/38	11/12	40/50	57/57	+++	+++
	Slender habitus	35/55	11/23	46/78	0/57	++	‐
	Kyphoscoliois	18/56	5/16	23/72	17/37	+	+
	Pectus excavatum/carinatum	19/56	11/19	30/75	3/37	+	+
**Other**	Hypertrichosis	0	0	0	29/35	‐	‐
	Gum hypertrophy	1/55	1/24	2/79	7/17	‐	‐
	Cadiac defects	3/55	1/24	4/79	6/35	+	‐
	Umbilical hernia	n.a	n.a	n.a	10/32	+	++

a Weight to length normal as typically length > P98.

b Typically mild (IQ 50–69) or borderline (IQ 70–75).

c Typically mild (IQ 50–69) or moderate (IQ 35–50).

No unbiased series of molecularly confirmed patients available; +++75–100%; ++ 25–75%; + 5–25%; ‐ 0–5%.

WV, wide ventricles; C, *Corpus callosum* underdevelopment; BA, brain atrophy; CM, chiari malformation.

**Table 2:** Spectrum of *NFIX* mutations in 42 presently reported patients and those reported in literature

Patient/Reference	cDNA	Protein	Location	Type of mutation	Inheritance	CADD PHRED score^b^
P1	c.[28‐1G > A;28‐12T > A;28‐13T > A]	p.Asp10Profs*5	Intron 1	Splicing	De novo	25
P2	c.43_49dup	p.(Glu17Valfs*31)	Exon 2	Frameshift	De novo	31
P3	c.59T > C	p.(Leu20Pro)	Exon 2	Missense	ND	26
*Klaassens 2015*	*c.90_99del*	p.(Trp30Cysfs*24)	Exon 2	Frameshift	De novo	32
P4	c.92T > C	p.(Phe31Ser)	Exon 2	Missense	De novo	26
*Martinez 2015*	*c.112C > T* ^1^ *published as c.136C > T*	p.(Arg38Cys) ^1^published as p.Arg46Cys	Exon 2	Missense	De novo	34
P5	c.112C > T	p.(Arg38Cys)	Exon 2	Missense	De Novo	34
P6	c.113G > T	p.(Arg38Leu)	Exon 2	Missense	ND	32
P7	c.142del	p.(Met48Cysfs*9)	Exon 2	Frameshift	De novo	28
P8	c.154_155insT	p.(Glu52Valfs*67)	Exon 2	Frameshift	De novo	33
*Priolo 2012*	*c.157_177del*	p.(Glu53_Glu59del)	Exon 2	In frame deletion	De novo	16
P9	c.157_177del	p.(Glu53_Glu59del)	Exon 2	In frame deletion	De novo	16
*Martinez 2015*	*c.161G > C* ^1^ *published as c.185G > C*	p.(Arg54Pro) ^1^published as p.Arg62Pro	Exon 2	Missense	De novo	31
*Yoneda 2012*	*c.179T > C*	p.(Leu60Pro)	Exon 2	Missense	De novo	26
P10	c.182T > C	p.(Leu61Pro)	Exon 2	Missense	De novo	26
P11	c.187G > T	p.(Glu63*)	Exon 2	Nonsense	De novo	39
*Gurrieri 2015*	*c.191del*	p.(Lys64Serfs*30)	Exon 2	Frameshift	De novo	27
P12	c.200_201dup	p.(Lys68Serfs*27)	Exon 2	Frameshift	De novo	31
P13	c.248T > G	p.(Ile83Ser)	Exon 2	Missense	De novo	28
*Oshima 2017*	*c.269dup* ^1^ *published as c.290_291insA*	p.(Asp90Glufs*29) ^1^published as p.Asp98GlyfsX29	Exon 2	Frameshift	De novo	31
P14	c.280A > C	p.(Thr94Pro)	Exon 2	Missense	ND	25
P15	c.294del	p.(Phe99Serfs*36)	Exon 2	Frameshift	De novo	28
P16	c.315_316insGGT	p.(Leu105_Ser106insGly)	Exon 2	In frame insertion	De novo	16
P17	c.317C > T	p.(Ser106Phe)	Exon 2	Missense	ND	28
P18	c.322_323delinsA	p.(Pro108Thrfs*27)	Exon 2	Frameshift	De novo	33
P19	c.325G > T	p.(Asp109Tyr)	Exon 2	Missense	De novo	29
P20	c.328C > T	p.(Gln110*)	Exon 2	Nonsense	De novo	37
P21	c.337A > G	p.(Lys113Glu)	Exon2	Missense	De novo	26
P22	c.343C > T	p.(Arg115Trp)	Exon 2	Missense	De novo	34
*Jezela‐Stanek 2016*	*c.343C > T* a *published as c.367C > T*	p.(Arg115Trp)^a^ published as p.Arg123Trp	Exon 2	Missense	De novo	34
P23	c.346C > G	p.(Arg116Gly)	Exon 2	Missense	De novo	27
P24	c.347G > A	p.(Arg116Gln)	Exon 2	Missense	ND	30
P25	c.347G > A	p.(Arg116Gln)	Exon 2	Missense	De novo	30
*Gurrieri 2015*	*c.347G > C*	p.(Arg116Pro)	Exon 2	Missense	De novo	29
P26	c.358delC	p.(Leu120Cysfs*15)	Exon 2	Frameshift	De novo	33
*Yoneda 2012*	*c.362G > C*	p.(Arg121Pro)	Exon 2	Missense	*Maternal*	30
*Gurrieri 2015*	*c.373A > G*	p.(Lys125Glu)	Exon 2	Missense	De novo	26
*Lu 2017*	*c.373G > T*	p.(Lys125Gln)	Exon 2	Missense	De novo	26
P27	c.383G > A	p.(Arg128Gln)	Exon 2	Missense	ND	32
P28	c.408delinsTT	p.(Lys136Phefs*3)	Exon 2	Frameshift	ND	31
P29	c.412_413delinsG	p.(Lys138Glyfs*73)	Exon 2	Frameshift	De novo	28
P30	c.444G > C	p.(Glu148Asp)	Exon 2	Missense	De novo	25
P31	c.463C > T	p.(Gln155*)	Exon 2	Nonsense	De novo	37
P32	c.499C > A	p.(His167Asn)	Exon 2	Missense	De novo	27
P33	c.520G > T	p.(Glu174*)	Exon 2	Nonsense	De novo	38
P34	c.542dup	p.(Tyr181*)	Exon 2	Frameshift	De novo	34
*Malan 2010*	*c.568C > T*	p.(Gln190*)	Exon 3	Nonsense	De novo	40
P35	c.694C > T	p.(Gln232*)	Exon 4	Nonsense	*Maternal mosaic*	40
P36	c.694C > T	p.(Gln232*)	Exon 4	Nonsense	De novo	40
P37	c.759C > G	p.(Tyr253*)	Exon 5	Nonsense	De novo	37
P38	c.779C > G	p.(Thr260Ser)	Exon 5	Missense	De novo	13
P39	c.859dup	p.(Glu287Glyfs*5)	Exon **6**	Frameshift	De novo	35
*Klaassens 2015*	*c.1012C > T*	p.(Gln338*)	Exon 7	Nonsense	De novo	42
P40	c.1055_1064del	p.(Pro352Leufs*46)	Exon 7	Frameshift	De novo	36
P41	c.1116del	p.(Ser373Profs*28)	Exon 8	Frameshift	De novo	34
P42	c.1117del	p.(Ser373Profs*28)	Exon 8	Frameshift	De novo	35

All the variants refer to main transcript and major isoform of *NFIX* gene) (NM_002501.3) (Q14938). Nomenclature was checked using Mutalyzer website: https://www.mutalyzer.nl/ND, not determined (due to unavailability of material from both parents).

a Mutations previously published with reference to another isoform (NM_001271043.2) that encodes a protein with eight additional amino acids at the N‐terminus.

b CADD (https://cadd.gs.washington.edu/) v1.3 PHRED‐like (‐10*log10(rank/total)) scaled C‐score: ranking a variant relative to all possible substitutions of the human genome (8.6 × 10^9). A scaled C‐score ≥10: variant is belongs to 10% most deleterious variants; C‐score ≥20: variant belongs to 1% most deleterious variants.

All variants have been submitted to: https://databases.lovd.nl/shared/genes/NFIX.

### 3.1 Phenotype

All patients presented with typical facial features of Malan syndrome (Figure 1; Supporting Information Figure 7). Notably, no patient had facial features typical for Sotos or Weaver syndromes. The phenotypes of the four patients (patients 39–42) with variants in the 3′end of *NFIX* were not different from the phenotype of patients with variants in other *NFIX* domains. Additional noteworthy data include two patients (patient 1 and 21) conceived via Artificial Reproduction Technique; underdeveloped optic nerves in patients 6, 7, 15, 21, 23, 24, 26, 29, 35, 37, 40, 41, and 42; periventricular nodular heterotopias (patients 7 and 23); highly arched palate and dental crowding (patients 5, 29, 33, 37, and 44); sparse hair (patients 24, 25, and 38); loose and soft skin of their soles (patients 25 and 39); and facial asymmetry (patients 2 and 6). Problems observed in individual patients were an abnormal pelvic bone morphology (patient 3); right coxa valga deformity and cox arthritis, cataract, and mitral valve prolapse (patient 5); absent earlobes (patient 6); mild restriction of elbow and knee movements (patient 13); camptodactyly (patient 14); contractures of his hands in early adulthood (patient 19); marked brachycephaly (patient 20); periventricular gliosis (patient 21); plagiocephaly (patient 22); contractures of the hamstrings, poor peripheral vision, atrial septal defect, and increased susceptibility for infections (patient 23); large but normally functioning kidneys (patient 24); a supraorbital cavernous hemangioma and choroid fissure cyst (patient 26); curved tibiae (patient 28); central obstructive apnea, a flaccid larynx, talipes and bilateral hydroceles (patient 34); three hemangiomas, a small optic chiasm, microcystic pineal gland, and cortical vision impairment (patient 35); hemiplegic migraine headaches (patient 37); postural hypotension (patient 38); grade IV hydronephosis due to bilateral proximal ureteral stenosis, an inguinal hernia, and brittle toenails (patient 39); gall bladder agenesis, intestinal malrotation, pulmonary valve stenosis, and severe dilatation of the pulmonary trunk (patient 42); and a sarcoma of the fifth rib at age 9 years (patient 43).

### 3.2 Genotype

Molecular results are listed in Table 2, and their distribution over the gene is illustrated in Figure 2. We found 18 missense and seven nonsense variants,14 small deletions and insertions/duplications causing frameshift and premature termination codons, two in‐frame insertions/deletions, and a complex de novo change at the splice acceptor region of exon 2 consisting of three nucleotide changes (c.28‐1G > A, c.28‐12T > A and c.28‐13T > A). Parents were negative for all three variants and paternity was confirmed.

Most variants were in exon 2. Only three variants (missense variants p.Arg38Cys and p.Arg115Trp; in frame deletion p.Glu53_Glu59del) had been previously reported (Jazela‐Staneck et al., 2016, Martinez et al., 2015, Priolo et al., 2012). The remaining variants are novel. We found two missense variants involving residue Arg116 (p.Arg116Gly; p.Arg116Glu) in three patients (Patients 23, 24, and 25). A different missense change was reported previously (p.Arg116Pro) (Gurrieri et al., 2015). All observed missense variants affected amino acids, highly conserved in orthologous and paralogous NFI proteins except for a change of Thr‐260, which is conserved in the CTF‐NFI domain of NFIX only (Supporting Information Figure 2). All variants were absent from 1000 genomes, ExAC, and gnomAD databases. In silico analysis of *NFIX* variants using CADD v1.3 PHRED‐like C‐score suggested that all variants are deleterious (Table 2). Only seven Malan syndrome‐associated variants are in exons 5–8 outside of the region coding for the N‐terminal DNA binding and dimerization domain. Of these, one is a missense, two are nonsense, and four are frameshift variants. These patients presented all a typical Malan phenotype (Supporting Information Table 1; Figure 1 a4, a5, b3, c1, e3, e6; Klaassens et al., 2015).

All variants for which parents were checked (*n* = 35) were de novo except for patient 35, in whom the mother was found to be mosaic for the *NFIX* variant in blood using WES (allele count abnormal in six of the total 44 reads [14%]). She was phenotypically normal, height was 165.1 cm, and had normal intelligence. As a child she had learning difficulties requiring individualized educational program and as an adult she had an anxiety disorder.

### 3.3 RNA analysis

RT‐PCR experiments were performed on RNA extracted from fibroblasts of two individuals. In patient 1 harboring the complex de novo variant c.[28‐1G > A;28‐12T > A;28‐13T > A] at the splice acceptor of exon 2 the consequence of this change was investigated by sequencing of cDNA. Thereby it could be demonstrated that the variant leads to utilization of an ectopic acceptor splice site, which resulted in an inclusion of 10 nucleotides of intron 1 into the mRNA that predicted a frameshift and premature stop codon (p.Asp10Profs*5) (Supporting Information Figure 3).

In patient 41 carrying c.1116del in exon 8, cDNA sequence analysis using oligonucleotide primers specific for different protein‐coding isoforms showed that the mutant allele was almost absent in the mRNA, thereby indicating degradation by NMD (Supporting Information Figure 4).

## 4 DISCUSSION

We report here on a series of 45 individuals with molecularly confirmed Malan syndrome, and compare the findings to those of 35 previously reported individuals (Supporting Information Tables 1–3). This has allowed us to refine the major characteristics of Malan syndrome.

Malan syndrome is known as an overgrowth syndrome. However, the combined data from previously published and presently reported cases reveals that birth weight is above the 2 SDS in only a minority of patients, but birth weight is above the mean in ∼90% of patients. Head circumference is > 2 SDS in 37% of newborns. Postnatal height and head size are typically > 2 SDS but height may decrease with age as of 13 adults only six still have a height > 2 SDS. Head circumference remained > 2SD in 10/13 adults. Bone age is advanced 1 year or more in 80% of patients in whom this was evaluated but data are available from only 50 out of 79 patients, with likely ascertainment bias as this is more frequently determined in those who show overgrowth. The growth pattern in Malan syndrome resembles the growth pattern in Sotos syndrome in which initial overgrowth is very common but adult height is above 2 SDS in only one‐third of individuals (Tatton‐Brown et al., 2013).

Intellectual disability is invariably present, but detailed studies are lacking. Preliminary results of ongoing studies in the presently reported group of individuals indicate that severity is varying from moderate to severe, and exceptionally mild intellectual disability is present. Independently, several referring physicians have indicated that their patients demonstrate anxieties and are extremely sensitive to noise, which seems to worsen their anxious behavior. In our series, anxieties were mentioned spontaneously by referring clinicians in the item “Behavioral characteristics” in 52% of individuals. The behavior may culminate in crises during which patients may become aggressive to themselves and to others. More detailed cognitive and behavioral studies are in progress to substantiate the behavior using dedicated psychological instruments.

Neuro‐imaging yielded normal results in most patients. If abnormalities were present, then these were usually nonspecific findings such as enlarged ventricles and a small callosal body. However, cortical dysplasias and periventricular nodular heterotopias do occur occasionally as well, as has been previously reported (Klaassens et al., 2015). Seizures and/or EEG abnormalities occurred in 18% of the presently reported patients and 27% of all known patients.

The most characteristic facial findings include a long or triangular face, prominent forehead, depressed nasal bridge, deeply set eyes, down‐slanting palpebral fissures, short nose with anteverted nares and upturned tip, long philtrum, small mouth that is often held open, with a thin upper vermillion in a cupid bow shape, an everted lower lip, and a prominent chin (Figure 1). With age the face seems to become more elongated, the chin becomes more prominent, skin folds deeper, and the mouth tends to become more open (Supporting Information Figure 7c). Visual problems are common (76%) but often limited to nonspecific abnormalities such as strabismus, myopia, and hypermetropia, and nystagmus is uncommon. Thirteen of the presently reported individuals had variable degrees of underdeveloped optic nerves, which has also been reported in three earlier reported patients (Dolan et al., 2010; Priolo et al., 2012). These individuals had both point variants and deletions of *NFIX*. Underdeveloped optic nerves have also been noticed in Marshall‐Smith syndrome (Shaw et al., 2010). Skeletal anomalies are frequent, especially a slender habitus (59%) together with long hands (60%) (Supporting Information Figure 7b), sometimes described as resembling Marfan syndrome. Other signs such as scoliosis, pectus carinatum, or excavatum occur at varying frequencies (Table 1).

When comparing characteristics of the 56 individuals with Malan syndrome caused by *NFIX* point mutations to the 24 individuals with deletions of *NFIX* and a variable number of other genes, there were no significant differences in growth pattern, cognitive impairment, facial characteristics, or skeletal manifestations. Individuals with an *NFIX* containing microdeletion demonstrated seizures and EEG abnormalities more frequently (11/24 in deleted patients vs. 10/56 in patients with point variants). The increased prevalence of seizures/epilepsy may be explained by the presence of a contiguous gene disorder. Deletions involving *CACNA1A* that is located 109 kb from *NFIX* seem to play a particular role. Of the 14 individuals in whom the deleted region included *CACNA1A*, 10 developed seizures. In contrast, only one patient has been reported with seizures in whom the deletion did not include *CACNA1A* (Jezela‐Stanek et al., 2016). Klaassens and coworkers (2015) reported a single patient with cyclical vomiting responsive to pizotifen and a literature patient who had episodic ataxia at age nine years. None of the present patients with *NFIX* deletions showed these symptoms.

We compared the findings in Malan syndrome to those reported in Marshall‐Smith syndrome (Shaw et al., 2010; Van Balkom et al., 2011), an allelic disorder caused by variants in *NFIX* that is postulated to result from a dominant‐negative action (Table 1; Figure 2). Individuals with Marshall‐Smith syndrome differ markedly in growth pattern, both prenatally and postnatally. Although the entity is often categorized among the overgrowth syndromes, 20% of newborns with Marshall‐Smith syndrome have in fact a birth weight below the 3^rd^ centile, none have macrocephaly, and almost all have growth parameters below the 3^rd^ centile postnatally. The wrongful assignment of Marshall‐Smith syndrome as an overgrowth disorder is caused by the seemingly advanced bone age of the distal extremities, which is a manifestation of the dysostosis in this disorder (Shaw et al., 2010). Cognitive impairment has been present in all known individuals with Marshall‐Smith syndrome (Van Balkom et al., 2011). Neuro‐imaging yields similar results in both entities. There are major differences in facial morphology due to the absence of macrocephaly and presence of proptosis, underdeveloped midface and small chin in Marshall‐Smith syndrome. Some facial findings such as a prominent forehead, short nose with anteverted nares, and everted lower lip are present in both entities, but generally the facial abnormalities in Marshall‐Smith syndrome are more pronounced. A slender habitus is uncommon in Marshall‐Smith and sternum abnormalities have been recorded only occasionally. Scoliosis is common in both entities, but only in individuals with Marshall‐Smith syndrome the scoliosis remains progressive also into adulthood, likely due to the marked osteopenia (RCH, personal observation). Hypertrichosis occurs only in Marshall‐Smith syndrome, and gingiva hypertrophy is almost completely limited to individuals with this entity. In only very few individuals with either entity one has considered the other diagnosis. In the present series, this occurred in patient 43. Malan syndrome and Marshall‐Smith syndrome seem to be two separate entities. However, the number of reported individuals remains small and with additional reports clues for a continuous spectrum may still be found.

Malan syndrome has been suggested to resemble Weaver syndrome and Sotos syndrome. We compared the three entities (Table I) and found some resemblances but also many differences. The growth pattern in these entities is similar: individuals with Sotos and Weaver syndrome are also frequently large for gestational age at birth, grow > 2 SDS in the first few years of life, but adult height falls typically within two SDS of the mean. Developmental delay is common in all three entities but in Malan syndrome there is typically moderate to severe intellectual disability, while Sotos and Weaver patients have typically mild to moderate disability, although there is a wide distribution of cognitive and functional abilities (Lane, Milne, & Freeth, 2016). Facially, individuals with Malan, Sotos, and Weaver syndrome share macrocephaly and a prominent forehead, and, in Sotos and Malan syndrome, a long face. The other facial signs differ between the three entities. We conclude that usually differentiating the three entities is possible by clinical evaluation alone, and that this differentiation is of major importance for adequate care and counseling of patients and their families. We propose that Malan syndrome should not be indicated as “Sotos syndrome type 2″ because of the clear differences between the two entities.

Together with the patient cohort presented here, a total of 51 different intragenic *NFIX* variants have been reported in 56 unrelated patients with Malan syndrome (Table 2). Twenty‐seven of the variants observed in Malan syndrome were nonsense, frameshift and splice site variants predicting premature stop codons mostly in the 5′ part of the mRNA. It is likely that these mutated mRNAs are cleared by nonsense‐mediated decay (NMD), thereby leading to haploinsufficiency (Lykke‐Andersen & Jensen 2015). Experimental evidence for this mechanism has previously been provided for a small number of variants causing Malan syndrome (Malan et al., 2010). Twenty‐three different missense variants have been identified, most of these (22 out of 23) are located in the DNA binding and dimerization domain. There is a predominant involvement of basic residues (in 12 of the 23 missense variants), and some have been altered more than once (at positions Arg38; Arg115; Arg116; Lys125) (Gurrieri et al., 2015; Jezela‐Stanek et al., 2016; Lu et al., 2017; Martinez et al., 2015; Priolo et al., 2012). We found a clustering of *NFIX* missense variants involving positively charged amino acids (at positions Lys 113, Arg115, Arg116, Arg121, Lys125, and Arg128) in a small region of 15 residues. Both arginine and lysine are frequently located in protein‐activating or binding sites, and their charge distribution is ideal for binding negatively charged groups (LaCasse et al., 1995; Yao et al., 2016). A putative nuclear localization (NLS) domain encompassing amino acids 36–50 of NFIX (RKRKYFKKHEKRMSK) is predicted by cNLS mapper (url: nls‐mapper.iab.keio.ac.jp/). This domain is completely overlapping with NLS1 in the paralogous NFIA protein. Previous studies on NFIA demonstrated that both NLS1 and NLS2 are required for the full translocation of the protein to the nucleus (Imagawa, Sakaue, Tanabe, Osada, & Nishihara, 2000). The domains are highly conserved in all NFI proteins and are located near the DNA binding domain (Imagawa et al., 2000). Three Malan syndrome‐associated missense variants are localized at or immediately adjacent tothe putative NLS. The NLS regions are usually located near DNA‐binding domains (LaCasse et al., 1995). Based on these suggestions it is plausible that the mutated proteins harboring missense variants may either fail to shuttle into the nucleus or bind DNA, thus explaining haploinsufficiency.

There are striking genotype‐phenotype correlations between Malan syndrome and Marshall‐Smith syndrome. Variants associated with Marshall‐Smith syndrome have exclusively been found in regions outside of those coding for the DNA binding and dimerization domain, especially in exons 6–8 and sometimes in exons 9 and 10 (Figure 2). For several Marshall‐Smith‐associated variants it has been demonstrated that mutant mRNA does not undergo NMD (Malan et al., 2010; Schanze et al., 2014). Previous evidence suggested that variants associated with Malan syndrome are instead restricted to the 5′ part of the gene encoding for the DNA binding and dimerization domain. This was indeed true for most of the novel variants reported in our study (Figure 2). In contrast, however, we identified four variants (in five individuals affected by Malan syndrome) that were located within the 3′ part of the gene (in exons 6, 7, and 8). Another variant in this part of the gene, p.Gln338*, has been reported previously in an individual with Malan syndrome (Klaassens et al., 2015). Their phenotypes (P39, P40, P41, P42 and Klaassens patient) do not differ in any way from those of other individuals with Malan syndrome. Alignment of the corresponding mutated NFIX proteins in Malan syndrome and Marshall‐Smith syndrome predicts that despite the overlap in the genomic positions of variants, the position of the translational stop codon strictly separates Marshall‐Smith syndrome—from Malan syndrome‐associated variants (Supporting Information Figures 5–6). We have also shown here for a Malan syndrome‐associated frameshift variant with the most 3′ position (c.1116del) that the mutant mRNA is almost absent, thus confirming the rule that haploinsufficiency is the mechanism underlying Malan syndrome. It cannot be excluded for Marshall‐Smith syndrome‐associated variants that aside from their ability to generate expressed mutant proteins with conserved DNA‐binding capacities, these proteins might also have specific functions mediated by their altered C‐terminus. However, the present analysis suggests that there is no motif shared by all Marshall‐Smith syndrome‐associated mutant NFIX proteins (Supporting Information Figures 5 and 6).

Malan syndrome is typically caused by a de novo mutational event (Table 2). However, one of the individuals presented here had a variant inherited from a mosaic mother who was phenotypically normal but who had some learning and behavioral issues that may point to a very mild presentation. There are two other reports of variants that have been inherited. In one, the p.Arg121Pro missense variant was inherited from a mother who presented with tall stature (no other clinical data provided) (Yoneda et al., 2012) and Nimmakayalu et al. (2013) reported two sisters with Malan syndrome and a partial *NFIX* deletion, suggesting germline mosaicism in one of the parents. Both parents were phenotypically normal and the deletion was not detected in their lymphocytes. These observations stress the importance of careful evaluation of parents and proper counseling about recurrence risks in families with Malan syndrome.

Individuals with Malan syndrome exhibit some symptoms reminiscent of Marfan or Marfan‐like syndromes caused by dysregulation of the TGF‐β pathway (slender body build, long fingers, scoliosis, and pectus formation). A possible pathophysiological link could be the interaction of NFI proteins with Sloan‐Kettering oncogene SKI (Tarapore et al., 1997), the protein mutated in Shprintzen‐Goldberg syndrome (Doyle et al., 2012). The SKI protein in turn regulates SMAD‐dependent TGF‐β signaling (Luo et al., 2004). Consistent with a possible relationship of Malan syndrome with Marfan‐like connective tissue disorders, one individual in the present series had a marked pulmonary artery dilatation, and two of the 15 individuals from the present series who underwent aortic evaluation, had an enlarged aorta (one at age 3 years). In the literature, two other individuals with Malan syndrome (one with a frameshift variant and one with a whole gene deletion) have been reported with aortic dilatation (Nimmakayalu et al., 2013; Oshima et al., 2017), and one individual with Marshall‐Smith syndrome has been reported with aortic root dilatation (Aggarwal, Nguyen, Rivera‐Davila, & Rodriguez‐Buritica, 2017). The true frequency, spontaneous course and clinical significance of aortic dilatations in Malan syndrome and Marshall‐Smith syndrome currently remains uncertain, but it seems prudent to evaluate all individuals with either syndromes for enlargement of the large vessels, until reliable data prove whether this is truly indicated or not.

We conclude that Malan syndrome is an overgrowth syndrome that presents in infancy and childhood, but overgrowth is less marked in adulthood as adult height is > 2 SDS in half of them. Macrocephaly remains present in 75%. There are significant and recognizable differences between Malan syndrome and other overgrowth syndromes, including Sotos and Weaver syndrome, and we therefore challenge the designation of “Sotos syndrome type 2″ for Malan syndrome. There may be an increased risk to develop aortic dilatation in Malan syndrome, but more data are needed to determine whether long‐term surveillance is indicated. There may be a specific behavioral phenotype, characterized especially by anxieties, but this requires further detailed studies that are presently in progress. Except for the occurrence of epilepsy, likely due to deletion of *CACNA1A*, there is no difference in phenotype between individuals with 19p13.2 microdeletions encompassing *NFIX* and those with intragenic mutations. *NFIX* variants that cause Malan syndrome are typically located in exons coding for the DNA binding and dimerization domain, with few exceptions. As for truncating variants elsewhere in *NFIX*, the mechanisms leading to haploinsufficiency rather than dominant‐negative effects for Malan syndrome‐associated frameshift variants in the 3′ exons of the gene are likely related to the position of the translational stop codon and clearance of the mutant mRNA by NMD.

## CONFLICTS OF INTEREST

The authors declare that there is no conflict of interest.

## Supporting information

Supporting information
