# Supplementary material for: Further delineation of Malan syndrome
Source: Hum Mutat. 2018 Jun 25;39(9):1226–37. doi: 10.1002/humu.23563 (PMC6175110; doi:10.1002/humu.23563)

Supporting information

Further delineation of Malan syndrome

Priolo M et al.

S1 Fig. Transcript isoforms of human *NFIX*. Graphical alignment of various known *NFIX* transcripts using the 11 exon transcript isoform ENST00000592199.5 as reference (top). Color coding illustrates sequences differing from this isoform by either using an alternative first exon (red or pink color) or by changing the reading frame in exon 10 (green color). This illustration demonstrates that resulting proteins may differ in the very N-terminal amino acids encoded by exon 1. The biological function and relevance of these variable N-termini of NFIX protein are unknown. Notably, the DNA binding and dimerization domain encoded by exons 2-4 is invariant in all isoforms except for transcript ENST00000588228.5 (printed in grey), whose expression and biological relevance is not clear. The alternatively spliced exon 7 is in frame, while alternative splicing of exon 9 leads to shifting of the reading frame resulting in a different C-terminus of the encoded proteins. Three very short transcript isoforms are also annotated as protein coding isoforms in the ENSEMBL database, but these have not been considered here, because the predicted gene products do not contain the typical functional domains defining a NFI protein and their biological function – if there is one anyway – remains unknown.
The isoform ENST00000592199.5 is identical to NM_002501.3 except for the lack of the alternatively spliced exon 9 in the latter. According to our own experiments the Δexon9 isoforms are more abundant in most tissues investigated including brain (see Suppl. Fig. S4). NM_002501.3 has been used as a reference in the majority of previous publications on *NFIX* variants. Due to the nature of those two transcripts all changes in exons 1-8 have the identical denomination.

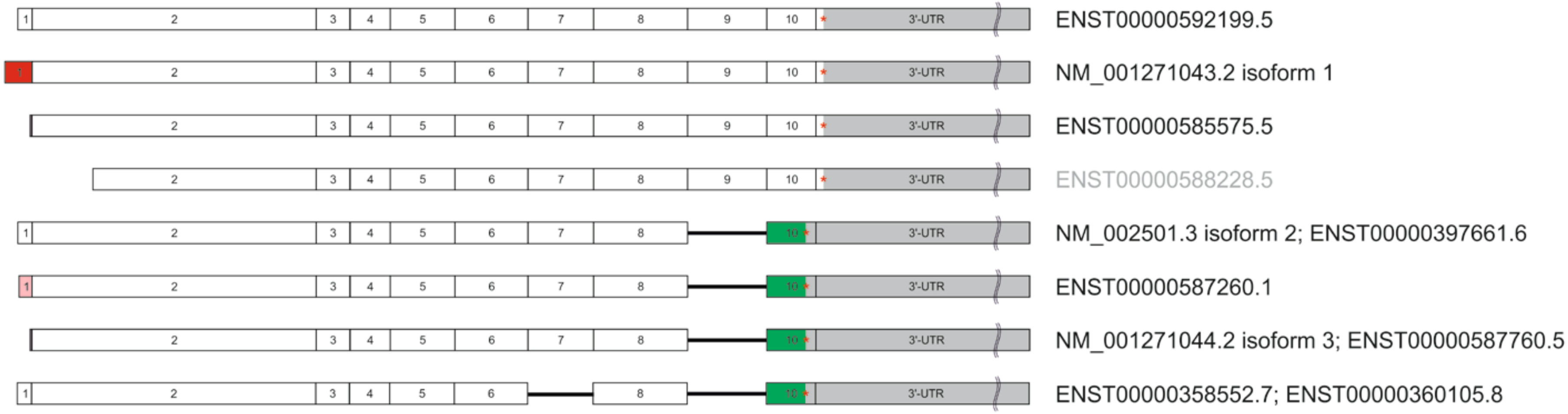

S2 Fig. Alignment of orthologous and paralogous NFI proteins: This alignment illustrates the conservation of amino acid residues affected by Malan syndrome-associated missense mutations (full alignment in S7 Fig.). Blue background color shows the putative DNA binding and dimerization domain of the gene and inside it, green color represents the MH1 (MAD homology 1) domain and the N-terminal DNA binding (DNAbd) domain. The orange colored box shows a small part of the CAAT-box transcription factor – nuclear factor I (CTF-NFI) domain containing the only missense mutation discovered in this domain. The observed missense changes are indicated on top of the alignment and the respective conserved amino acid is printed in red, while the amino acids affected by the in frame deletion E53_E59del are printed in blue. The putative nuclear translocation signal sequence (NLS1) is indicated as a black horizontal bar.

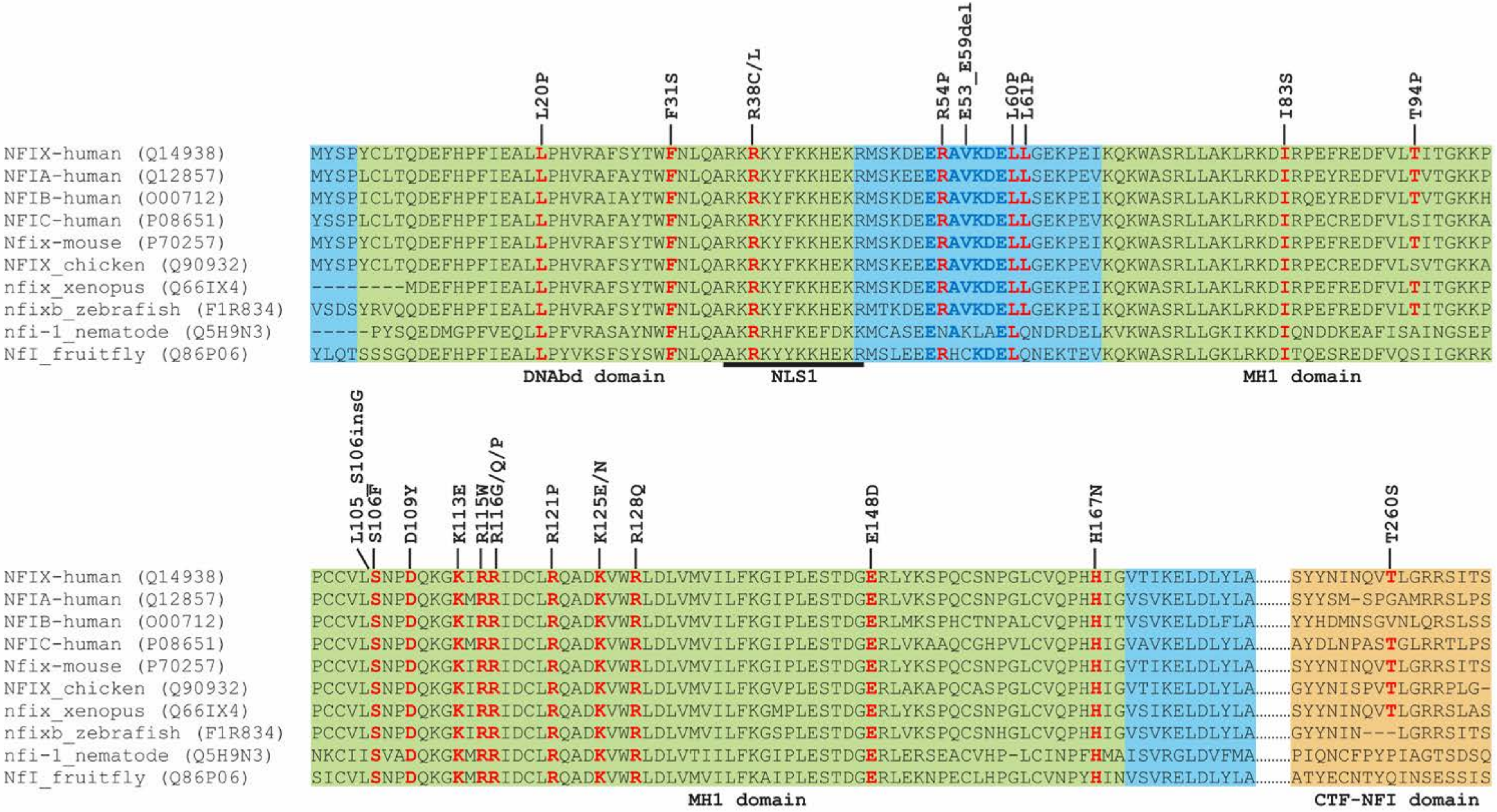

S3 Fig. Demonstration of altered splicing caused by a complex mutation in intron 1: Results of genomic and cDNA sequencing in patient 1 carrying the *de novo* mutation c.[28-1G>A;28-12T>A;28-13T>A] in intron 1 are shown. (**A**) Genomic sequence showing the three variants. The *cis*-phase of the three variants has been confirmed by visual evaluation of NGS read data using the Integrative Genomics Viewer (IGV; data not shown). The ag at the authentic splice acceptor is highlighted by a gree frame and the predicted new acceptor splice site is highlighted by a violet frame. Exonic nucleotides are printed in capitals. (**B**) cDNA sequencing from a normal individual and patient 1 (electropherograms from sequencing of the reverse strand are shown). The vertical lines indicate the exon 1 – exon 2 junction. The inserted 10 nucleotides from intron 1 into the mutant RNA are printed in red color.

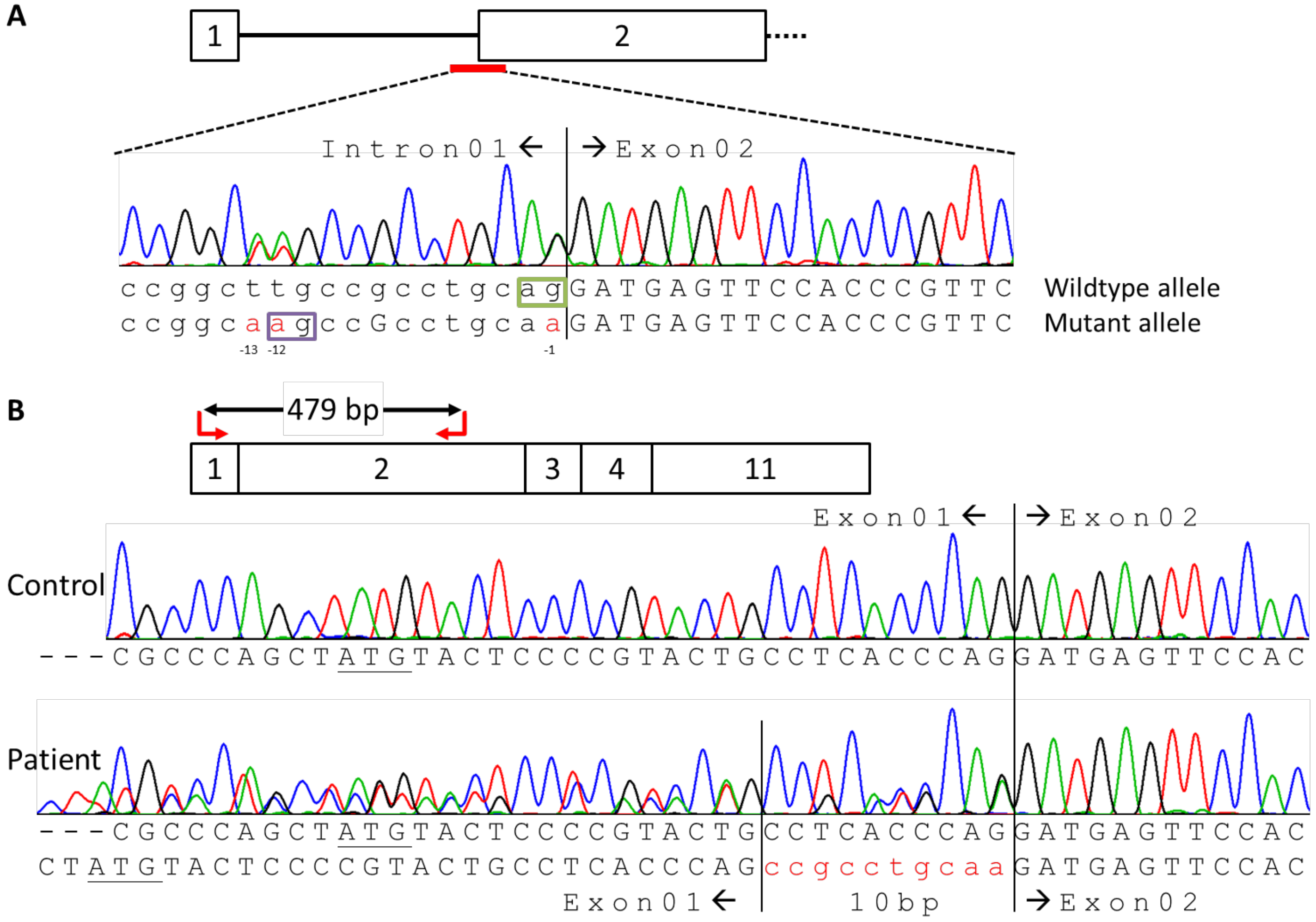

S4 Fig. The frameshift mutation c.1116del in exon 8 leads to NMD. RT-PCR and *NFIX* cDNA sequencing on RNA derived from fibroblasts of patient 41 with the heterozygous c.1116del variant. Four cDNA samples derived from fibroblast cultures from this patient were available (cDNA1-4) and one control fibroblast cDNA was used (co-cDNA). (**A**) Primer positions in exon 7 (forward) and 10 (reverse) allow the amplification of a cDNA fragment from the long *NFIX* isoform and an isoform lacking exon 9 (Δe9 isoform). The resulting agarose gel electropherogram demonstrates that fragment representing the Δe9 isoform is much more abundant in the fibroblast cDNA compared to the long isoform. (**B**) Primer positions in exon 7 (forward) and at the exon 8 – exon 9 junction (reverese) lead to specific amplification of the fragment from the long isoform. The resulting agarose gel electropherogram shows a distinct band at the expected size. (**C**) Sequence electropherograms obtained from sequencing of genomic DNA from the patient and of the RT-PCR products shown in (A) and (B), respectively. Genomic DNA shows the heterozygous frameshift variant, while in cDNA containing mainly the Δe9 isoform as well as from the long isoform only the wild type sequence is clearly visible. Only traces of the mutant allele may be appreciated (blue arrows). These findings indicate that mutant RNA, both from the long and from the Δe9 isoforms, is almost completely degraded by nonsense-mediated decay (NMD).

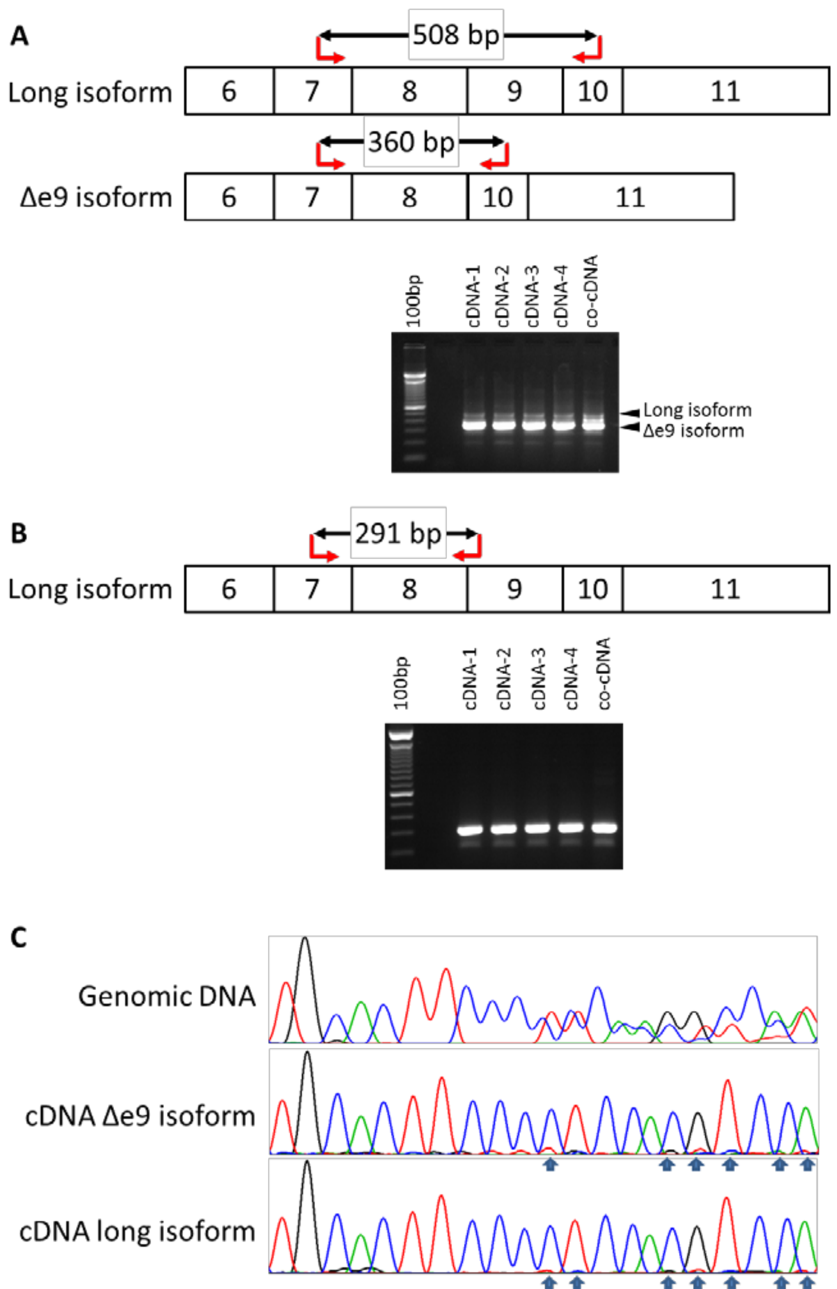

S5 Fig. Graphical presentation of a Clustal-Omega alignment of normal and mutated *NFIX* transcripts. (**A**) Wild type referring to the 11 exon transcript isoform ENST00000592199.5 (see Suppl. Fig. S1). (**B**) Marshall-Smith syndrome-associated mutated transcripts (all of them create a shift of the reading frame) and (**C**) Malan syndrome-associated transcripts carrying frameshift mutations in exons 6 and 8 are displayed. Deleted exons are indicated by a black horizontal bar. Numbers at the bottom of each transcript indicate the codon position of the shift of the reading frame. Shifting of the reading frame by -1/+2 (as for 1bp insertions) is indicated by blue color. Shifting of the reading frame by +1/-2 (as for 1bp deletions) is indicated by yellow color. The red asterisk marks the position of the predicted translational stop codon. As it can be appreciated, all Marshall-Smith syndrome-associated mutations create translational stop codons in exon 9, 10 or 11, while in Malan syndrome-associated frameshift mutations the stop codon occurs before exon 9 in this isoform. Marshall-Smith syndrome-associated mutations creating a shift of the reading frame by -1/+2 predict a common C-terminal tail of the encoded mutant protein, but mutations creating a shift of the reading frame by +1/-2 predict various different C-termini, thus not supporting a hypothesis of a specific abnormal function conferred by a uniform C-terminal domain of mutant proteins.

Specific coloring of mutations names encodes the references for the respective mutations:

green: Schanze et al., Hum Mutat 2014; 35:1092-1100.

light blue: Malan et al., Am J Hum Genet 2010; 87:189-198.

dark blue: Aggarwal et al., Eur J Med Genet 2017; 60:391-394.

violet: Martinez et al., Pediatr Res 2015; 78:533-539.

red: this report

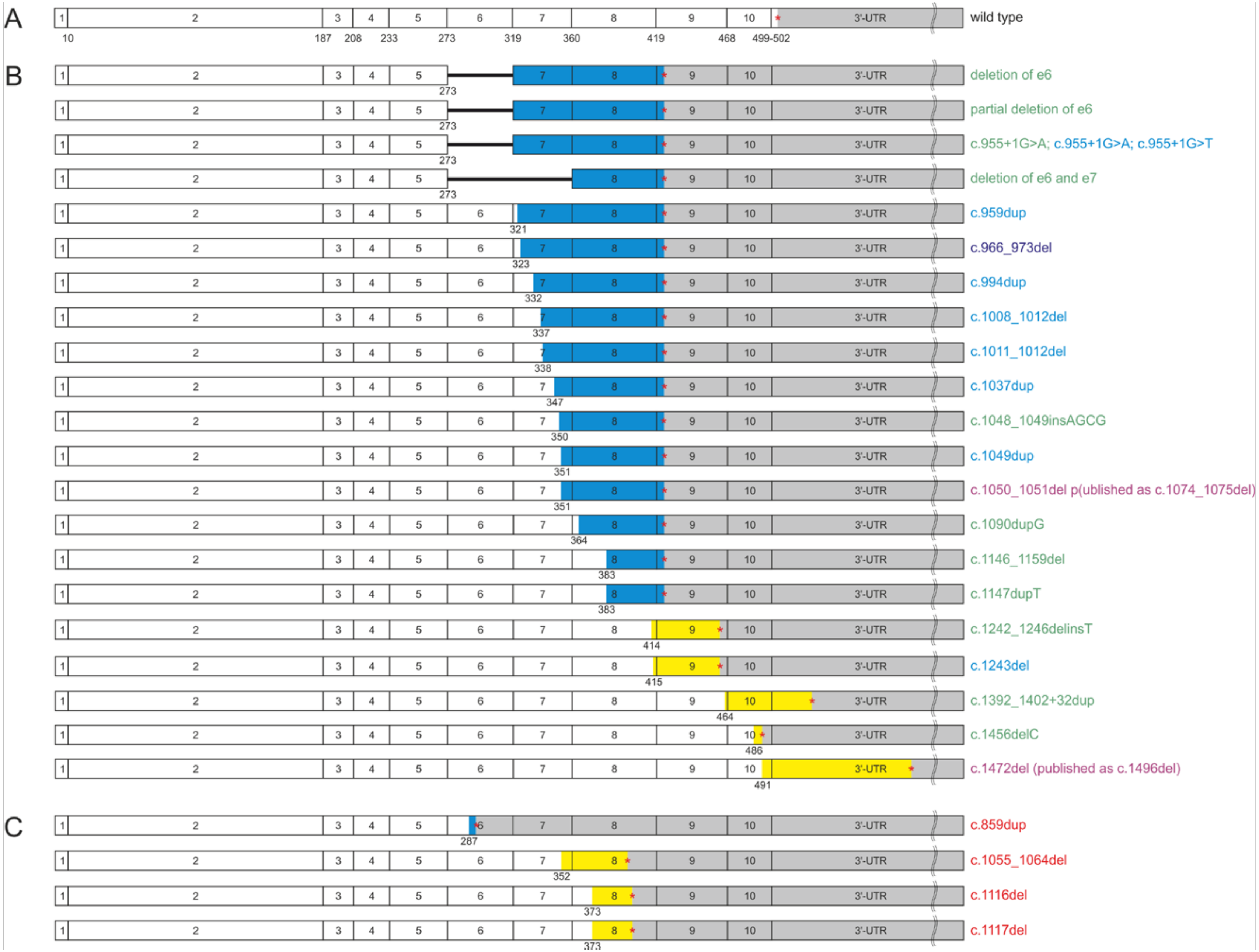
A
wild type
1 2 3 4 5 6 7 8 9 10 3'-UTR
10 187 208 233 273 319 360 419 468 499-502
B
deletion of e6
partial deletion of e6
c.955+1G>A; c.955+1G>A; c.955+1G>T
deletion of e6 and e7
c.959dup
c.966_973del
c.994dup
c.1008_1012del
c.1011_1012del
c.1037dup
c.1048_1049insAGCG
c.1049dup
c.1050_1051del p(ublished as c.1074_1075del)
c.1090dupG
c.1146_1159del
c.1147dupT
c.1242_1246delinsT
c.1243del
c.1392_1402+32dup
c.1456delC
c.1472del (published as c.1496del)
C
c.859dup
c.1055_1064del
c.1116del
c.1117del

S6 Fig. Graphical presentation of a Clustal-Omega alignment of normal and mutated NFIX transcripts (short isoform). (**A**) Wild type referring to the shorter 10 exon transcript isoform (NM_002501.3; Q14938; Δe9 isoform) lacking exon 9 of the long isoform ENST00000592199.5 (see Suppl. Fig. S1 and Suppl. Fig. S5 for comparison). (**B**) Marshall-Smith syndrome-associated mutated transcripts and (**C**) Malan syndrome-associated transcripts carrying frameshift mutations in exons 6 and 8 are displayed. Deleted exons are indicated by a black horizontal bar. Numbers at the bottom of each transcript indicate the codon position of the shift of the reading frame. Shifting of the reading frame by -1/+2 (as for 1bp insertions) is indicated by blue color. Shifting of the reading frame by +1/-2 (as for 1bp deletions) is indicated by yellow color. The red asterisk marks the position of the predicted translational stop codon. It can be appreciated that all Marshall-Smith syndrome-associated mutations create translational stop codons in the last exon of this isoform, while in the case of Malan syndrome-associated frameshift mutations the stop codon occurs before the last exon. For this isoform, which has been found to be the most abundant one in fibroblasts (Suppl. Fig. S4A), it is evident that Marshall-Smith syndrome-associated mutations cannot activate nonsense-mediated decay (NMD). No uniform C-terminal tail is predicted for Marshall-Smith syndrome-associated mutant proteins.

Specific coloring of mutations names encodes the references for the respective mutations:

green: Schanze et al., Hum Mutat 2014; 35:1092-1100.

light blue: Malan et al., Am J Hum Genet 2010; 87:189-198.

dark blue: Aggarwal et al., Eur J Med Genet 2017; 60:391-394.

violet: Martinez et al., Pediatr Res 2015; 78:533-539.

red: this report

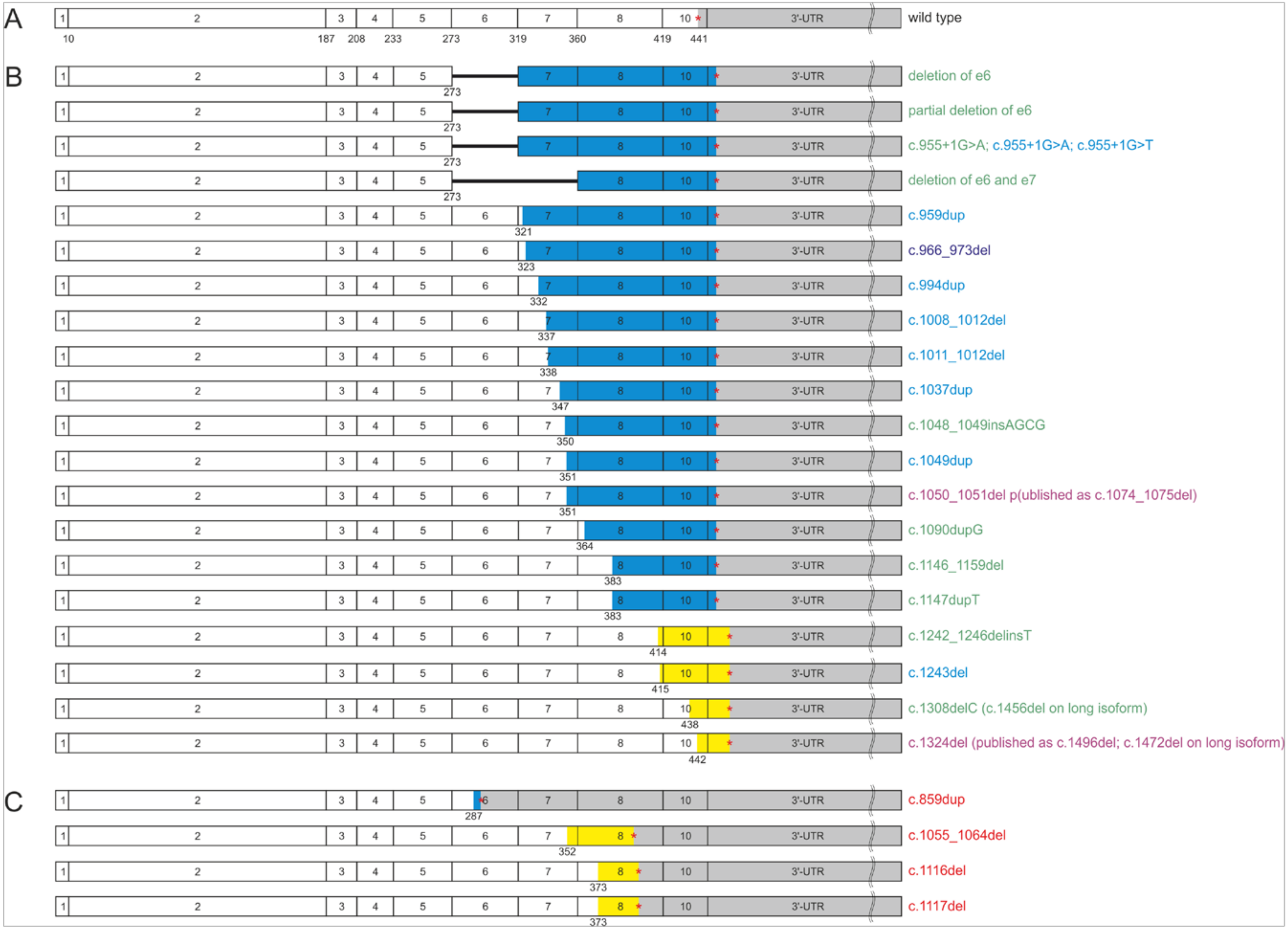

A
B
C
wild type
3'-UTR
10
187
208
233
273
319
360
419
441
deletion of e6
partial deletion of e6
c.955+1G>A; c.955+1G>A; c.955+1G>T
deletion of e6 and e7
c.959dup
c.966_973del
c.994dup
c.1008_1012del
c.1011_1012del
c.1037dup
c.1048_1049insAGCG
c.1049dup
c.1050_1051del p(ublished as c.1074_1075del)
c.1090dupG
c.1146_1159del
c.1147dupT
c.1242_1246delinsT
c.1243del
c.1308delC (c.1456del on long isoform)
c.1324del (published as c.1496del; c.1472del on long isoform)
c.859dup
c.1055_1064del
c.1116del
c.1117del

S7 Fig. Clustal-Omega alignment of orthologous and paralogous NFI proteins: Complete Clustsal-Omega v1.2.4 (https://www.ebi.ac.uk/Tools/msa/clustalo/) alignment of orthologous and paralogous NFI Uniprot (http://www.uniprot.org/; accession number given in brakets) proteins from caenorhabditis elegans: nfi-1 (Q5H9N3), drosophila melanogaster: NfI (Q86P06), homo sapiens: NFIA isoform 1 (Q12857), NFIB isoform 1 (O00712), NFIC isoform 1 (P08651), NFIX isoform 1 (Q14938), danio rerio: nfixb (F1R834), gallus gallus: nfix (Q90932), xenopus laevis: nfix (Q66IX4), and mus musculus: nfix (P70257).

```
CLUSTAL O(1.2.4) multiple sequence alignment

nfi-1_c_elegans        -------MEPHLKIDVSSASGSTT----TGA---TASTSEAPQDSQAQQTMPPPSSDWSNQFNSPEAVSPKANGIKCFS-----PYSQEDMGPFVEQLLPFVRASAYNWFHLQAAKRRHF
NfI_drome              ------------------------------------------------------------MFI-PETLRGCMIETDVSSYLQTSSSGQDEFHPFIEALLPYVKSFSYSWFNLQAAKRKYY
NFIA-human_iso1        -------------------------------------------------------------------------------MYSPLCLTQDEFHPFIEALLPHVRAFAYTWFNLQARKRKYF
nfixb_danio_rerio      XDSFAVPLPPRSFF----FSGQTFLFFSKGVKWVFETVGNIPRST----------MATEPFLVNEDPSRGPGIP-ECFVVSDSYRVQQDEFHPFIEALLPHVRAFSYTWFNLQARKRKYF
NFIX_chicken           -------------------------------------------------------------------------------MYSPYCLTQDEFHPFIEALLPHVRAFSYTWFNLQARKRKYF
nfix_xenopus           ---------------------------------------------------------------------------------------MDEFHPFIEALLPHVRAFSYTWFNLQARKRKYF
NFIX-human_iso1        -------------------------------------------------------------------------------MYSPYCLTQDEFHPFIEALLPHVRAFSYTWFNLQARKRKYF
NFIX-mouse             -------------------------------------------------------------------------------MYSPYCLTQDEFHPFIEALLPHVRAFSYTWFNLQARKRKYF
NFIB-human_iso1        ------------------------------------------------------------------------------MMYSPICLTQDEFHPFIEALLPHVRAIAYTWFNLQARKRKYF
NFIC-human_iso1        ------------------------------------------------------------------------------MYSSPLCLTQDEFHPFIEALLPHVRAFAYTWFNLQARKRKYF
                                                                                                               ::: **:* ***.*:: :*.**:*** **:::

nfi-1_c_elegans        KEFDKKMCASEENAKLAELQNDRDELKVKWASRLLGKIKKDIQNDDKEAFISAINGSEPNKCIISVADQKGKMRRIDCLRQADKVWRLDLVTIILFKGIPLESTDGERLERSEACVHP-L
NfI_drome              KKHEKRMSLEEERHCKDELQNEKTEVKQKWASRLLGKLRKDITQESREDFVQSIIGKRKSICVLSNPDQKGKMRRIDCLRQADKVWRLDLVMVILFKAIPLESTDGERLEKNPECLHPGL
NFIA-human_iso1        KKHEKRMSKEEERAVKDELLSEKPEVKQKWASRLLAKLRKDIRPEYREDFVLTVTGKKPPCCVLSNPDQKGKMRRIDCLRQADKVWRLDLVMVILFKGIPLESTDGERLVKSPQCSNPGL
nfixb_danio_rerio      KKHEKRMTKDEERAVKDELLGEKPEIKQKWASRLLAKLRKDIRPEFREDFVLTITGKKPPCCVLSNPDQKGKIRRIDCLRQADKVWRLDLVMVILFKGSPLESTDGERLVKSPQCSNHGL
NFIX_chicken           KKHEKRMSKEEERAVKDELLGEKPEVKQKWASRLLAKLRKDIRPECREDFVLSVTGKKAPCCVLSNPDQKGKIRRIDCLRQADKVWRLDLVMVILFKGVPLESTDGERLAKAPQCASPGL
nfix_xenopus           KKHEKRMSKDEERAVKDELLGEKPEIKQKWASRLLAKLRKDIRPEFREDFVLTITGKKPPCCVLSNPDQKGKIRRIDCLRQADKVWRLDLVMVILFKGMPLESTDGERLYKSPQCSNPGL
NFIX-human_iso1        KKHEKRMSKDEERAVKDELLGEKPEIKQKWASRLLAKLRKDIRPEFREDFVLTITGKKPPCCVLSNPDQKGKIRRIDCLRQADKVWRLDLVMVILFKGIPLESTDGERLYKSPQCSNPGL
NFIX-mouse             KKHEKRMSKDEERAVKDELLGEKPEIKQKWASRLLAKLRKDIRPEFREDFVLTITGKKPPCCVLSNPDQKGKIRRIDCLRQADKVWRLDLVMVILFKGIPLESTDGERLYKSPQCSNPGL
NFIB-human_iso1        KKHEKRMSKDEERAVKDELLSEKPEIKQKWASRLLAKLRKDIRQEYREDFVLTVTGKKHPCCVLSNPDQKGKIRRIDCLRQADKVWRLDLVMVILFKGIPLESTDGERLMKSPHCTNPAL
NFIC-human_iso1        KKHEKRMSKDEERAVKDELLGEKPEVKQKWASRLLAKLRKDIRPECREDFVLSITGKKAPGCVLSNPDQKGKMRRIDCLRQADKVWRLDLVMVILFKGIPLESTDGERLVKAAQCGHPVL
                       *:.:*:*  .**.    ** .:: *:* *******.*::***  : :* *: :: *..   *::*  *****:****************** :****. ********** :   *    *

nfi-1_c_elegans        CINPFHMAISVRGLDVFMANYLKDVDTKITLT--Y--------------PRND------ELSDT--VMVKQEPGEQTIVAPHAVLGTSSTHTRVWETNSERNAETIIITYDPQKACHTYF
NfI_drome              CVNPYHINVSVRELDLYLANFINTHNSINNTNTTFSTTPGVRSPHITMYNRSNDPNRKDEVADMKNDVVKQNPY------NGVVCNDIILATGVFSSQELWT-----LSKDLIL------
NFIA-human_iso1        CVQPHHIGVSVKELDLYLAYFVHAA-DSSQSES---------------------PSQP-SDADIKD----QPEN------GHLGFQDSFVTSGVFSVTELVR-----VSQTPIA------
nfixb_danio_rerio      CVQPHHIGVSVKELDLYLAYFVHTPAESGQSDN---------------------SNQQ-GEADIKP-----PPN------GHLSFQDCFVTSGVWNVAELVR-----VSQTPVA------
NFIX_chicken           CVQPHHIGVTIKELDLYLAFFVQAP-DSGQSDS---------------------SNPQ-GDADIKP-----LPN------GHLSFQDCFVTSGVWNVTELVR-----VSQTPVA------
nfix_xenopus           CVQPHHIGVSIKELDLYLAFFIHTP-DSGQSDA---------------------SNQ--TEVDIKP-----PLN------GHLSFQDCFVTSGVWNVTELVR-----VSQTPVA------
NFIX-human_iso1        CVQPHHIGVTIKELDLYLAYFVHTP-ESGQSDS---------------------SNQQ-GDADIKP-----LPN------GHLSFQDCFVTSGVWNVTELVR-----VSQTPVA------
NFIX-mouse             CVQPHHIGVTIKELDLYLAYFVHTP-ESGQSDS---------------------SNQQ-GDADIKP-----LPN------GHLSFQDCFVTSGVWNVTELVR-----VSQTPVA------
NFIB-human_iso1        CVQPHHITVSVKELDLFLAYYVQEQ-DSGQSGS---------------------PSHN-D----------PAKN------PPGYLEDSFVKSGVFNVSELVR-----VSRTPIT------
NFIC-human_iso1        CVQPHHIGVAVKELDLYLAYFVRER-DAEQSGS---------------------PRTG-MGSDQED--SKPITL------DTTDFQESFVTSGVFSVTELIQ-----VSRTPVV------
                       *::*.*: :::: **:::* ::.                                                                    : *:.  .        ::

nfi-1_c_elegans        GGRATLAQQSLSAGNTYMVNKTAVDNNFFNAKRSVLCLPPP-PIQNCFPYPIAGTSDSQQMDMSEDSNDGPSEKRSRDISSHDSPNSSTNDEVRRIVESGT--EKLVLGSSIWAAPGQFS
NfI_drome              ------------DES------NDINLNS-----SLIKRENVGATYECNTYQINSESSISA----AQSLVQSGSIAANPIPLGYSIDFI--DQ--RITQLSQSPLPRTEGPNGDSHDAN--
NFIA-human_iso1        ------------AGT------G---PNF-----SLSDLESS-SYYSMSPGAM--RRSLPS----TSST--SSTKRLKSVE--DEMDSPGEEP--FYTGQGRSPGSGS-QSSGWH---E--
nfixb_danio_rerio      ------------TAT------G---PNF-----SLADLDSPG-YYNI---NLG-RRSITS----TPST--SSNK-RKSID-DSEMESPVDDV--FYSG--RSPAPGASQPSGWP---N--
NFIX_chicken           ------------TAS------G---PNF-----SLADLESPGGYYNISPVTLG-RRPLG-----PPTA--SGPKRPKALD-EGDLEGPGDDV--FYSGPGRSPAPGSSQ-GPWG---G--
nfix_xenopus           ------------TSS------G---PNF-----SLADLESP-SYYNINQVTLG-RRSLAS----PPSS--SSTKRPKSLD-DSEMESPVDDV--FYPGTGRSPAAGGSQANVWP---N--
NFIX-human_iso1        ------------TAS------G---PNF-----SLADLESP-SYYNINQVTLG-RRSITS----PPST--STTKRPKSID-DSEMESPVDDV--FYPGTGRSPAAGSSQSSGWP---N--
```

```
NFIX-mouse             ------------TAS------G---PNF-----SLADLESP-SYYNINQVTLG-RRSITS----PPST--SSTKRPKSID-DSEMESPVDDV--FYPGTGRSPAAGSSQSSGWP---N--
NFIB-human_iso1        ------------QGT------G---VNF-----PIGEIPSQP-YYHDMNSGVNLQRSLSS-------P--PSSKRPKTISIDENMEPSPTGD--FYPS----PSSPAAGSRTWH---E--
NFIC-human_iso1        ------------TGT------G---PNF-----SLGELQGHL-AYDLNPASTGLRRTLPS----T-SS--SGSKRHKSGSMEEDVDTSPGGD--YYTS----PSSPTSSSRNWT---E--
                                     .           *       :                                         .      . :

nfi-1_c_elegans        RTQQNQGAPGTSRQVR-PLPDFQSQDSARSPG--AFRSTAKPVCRMTVNTGNHGDVGVVVVDERNREHVIHA--QHIV-NALSSLRTTPSMRESPVGRKRMHPHTSNSFEFLNCNQEMNK
NfI_drome              KIDQSSSPPITSTASDNGTSSFSLIVRATDSSIEDPKTSPISLHRYTSLSSRT--LAQAL--PQHSCSLLHSSST----------SPTN--------TLYYPQHNNTRPSQTTSV----E
NFIA-human_iso1        VEP-GMPSPTTLK--KSEKSGFSSPSPSQT----SSLGTAFTQHHRPVITGPR-AS------PHATPSTLHFPTSPIIQQ-PGPYFSHP--------AIRYH------------------
nfixb_danio_rerio      DVD---AVQHHLA--SMQER-------------------KIKHESDGVQFPYHAGS------PRASASSLHFPSPSLIQQ-SSPYFTHP--------TIRYH-HH---------------
NFIX_chicken           DVD---TSPATLK--KSGKLDFCSALSGHA----ASPRMAFGHHPLPVLAGVRPA------------SALHFPSGSLLPQ-SGPYFAHP--------TIRYH------------------
nfix_xenopus           DVD---TGPGSLK--KPGKLDFCSALSSQT----SSSRMAFTHHPLPVLAGVRPGS------PRASASALHFPSTSIIQQ-SSPYFTHP--------TIRYHHHH---------------
NFIX-human_iso1        DVD---AGPASLK--KSGKLDFCSALSSQG----SSPRMAFTHHPLPVLAGVRPGS------PRATASALHFPSTSIIQQ-SSPYFTHP--------TIRYH-HH---------------
NFIX-mouse             DVD---AGPASLK--KSGKLDFCSALSSQG----SSPRMAFTHHPLPVLAGVRPGS------PRATASALHFPSTSIIQQ-SSPYFTHP--------TIRYH-HH---------------
NFIB-human_iso1        RDQ-DMSSPTTMK--KPEKPLFSSASPQDS----SPRLSTFPQHHHPGIPGVAHSVISTR--TPPPPSPLPFPTQAILPPAPSSYFSHP--------TIRYPPHL---------------
NFIC-human_iso1        DMEGGISSPVKKT--EMDKSPFNSPSPQD-----SPRLSSFTQHHRPVIAVH---SGIAR--SPHPSSALHFPTTSILPQTASTYFPHT--------AIRYPPHL---------------
                                                                                             :

nfi-1_c_elegans        NEGALGSDIS-PTHTAVSNLIS---RESSGYM-----ASPTKFTTAR------GDTTSFSKIFQKIEEKHLQHNQPSTSYCNSQIQPPILSSKPVDSSVKLIAPVAVKPIMSGCNSIIPS
NfI_drome              EQNRVGSDKYSTEPQDISDFVTYVCQDTSHTTTITGSAENHSFQHSHTLPQGHGHSPHF-QIHQQLRSAKL-TTSPSTHYHSTMLPPM-L--PPMARPVAIIRS-------SSDLTLVQS
NFIA-human_iso1        ------------PQETLKEFVQLVCPDAGQQAGQVGFLNP------NGSSQG--K------V-------------HNPFLPTPMLPPPPP--PPMARPVPLPV-----------------
nfixb_danio_rerio      -----------PGQDPLKEFVQFVCADGSGQASGQ----H------TPRQAP--Q------L-------------P------------------TGLSAS--------------------
NFIX_chicken           -----------HGQDSLKDFVQFVCADGAAQGPQH----S------QRPA--------------------------------------PP--LPPALSAS--------------------
nfix_xenopus           -----------HTQDSLKEFVQFVCSDTSGQGSGQ----H------SQ-------------------------------R------LPPP--GPTGLS----Q-----------------
NFIX-human_iso1        -----------HGQDSLKEFVQFVCSDGSGQATGQ----P------NGSGQG--K------V-------------PGSFL------LPPP--PPVARPVPLPM-----------------
NFIX-mouse             -----------HGQDSLKEFVQFVCSDGSGQATGQ----P------NGSGQG--K------V-------------PGSFL------LPPP--PPVARPVPLPM-----------------
NFIB-human_iso1        -----------NPQDTLKNYVPSY-DPSSPQTS---------------------------------------------------------------------------------------
NFIC-human_iso1        -----------NPQDPLKDLVSLACDPASQQPGPL-----------NGSGQL--K------M-------------PSHCLSAQMLAPPPP--GLPRL----AL-----------------
                                       :.: :       .

nfi-1_c_elegans        PITTPRITPSFRMLEDDSLINVL-----GQLAH--SNDGTTLNDSFIQHLIDT-------NSRSPLLSSGNAFSALSMGAVSGLVPGNSIHRPDSSA--------------------SNG
NfI_drome              PPTSLPLTAQSTSLNDNSCIAKHSLPTDNRLASNLDNRNSPNNTDVVSQHEMTGTASPQPQSHTPTLASPQSY--LDPGRCKLLSAGSNIGRITTQMYPSSNNREYFNHFHSQPTSLLGY
NFIA-human_iso1        PDTKPPTTST-----EGGA-ASPTSPTY------STPSTSPANR-FVSVGPRDPSFVNIPQ------QTQSWY--LG-------------------------------------------
nfixb_danio_rerio      ---------------------DHGTTTF--------------------------------------------------------------------------------------------
NFIX_chicken           ---------------------DPATATF--------------------------------------------------------------------------------------------
nfix_xenopus           --------------------LDHETPTF--------------------------------------------------------------------------------------------
NFIX-human_iso1        PDSKSTSTAP-----DGAA-LTPPSPSF------ATTGASSANR-FVSIGPRDGNFLNIPQ------QSQSWF--L--------------------------------------------
NFIX-mouse             PDSKTTSTAP-----DGAA-LTPPSPSF------TTTGASSANR-FVGIGPRDG------------------------------------------------------------------
NFIB-human_iso1        ---------------------------------------------------------------------QSWY--LG-------------------------------------------
NFIC-human_iso1        PPATKPATTS-----EGGA-TSPTSPSY------SPPDTSPANRSFVGLGPRDPAGI---Y------QAQSWY--LG-------------------------------------------

nfi-1_c_elegans        SNSLGVAMGLAVPQNIALAVQQTQNAMSPLHQIRVSVGAPPACSPSSSNSSLGAANQAPVSNTPQDPNAPKLP-------TDFSHALRNEKK------
NfI_drome              AGSISTMSGVISPTDLTLYS------------------APMAVSRSSTRTRWNEEEH---NVIPQSASSTNMDNTQVILMEDSTGRYIDEYSSRDYVS
NFIA-human_iso1        --------------------------------------------------------------------------------------------------
nfixb_danio_rerio      --------------------------------------------------------------------------------------------------
NFIX_chicken           --------------------------------------------------------------------------------------------------
nfix_xenopus           --------------------------------------------------------------------------------------------------
NFIX-human_iso1        --------------------------------------------------------------------------------------------------
NFIX-mouse             --------------------------------------------------------------------------------------------------
NFIB-human_iso1        --------------------------------------------------------------------------------------------------
NFIC-human_iso1        --------------------------------------------------------------------------------------------------
```

Table Ia. Newly Reported Patients with Malan Syndrome and Variants in *NFIX*.

**ID**		1	2	3	4	5	6	7	8	9	10	11	12	13	14	15
**Gender**		M	F	F	M	M	M	M	F	M	F	F	M	F	M	F
**Age**		12y	16y	20y	14y	13y	42y	3y	31y	12y	15y	14y	9y	12y	14y	11yr
**Prenatal growth**	Weight>2SDS	+	-	-	-	-	?	-	-	-	-	+	+	-	-	-
	Length>2SDS	+	?	-	-	?	?	-	+	-	?	+	?	?	-	?
	OFC >2SDS	+	?	?	-	?	?	-	+	+	?	+	?	?	+	?
**Postnatal growth**	Height >2SDS	+	-	+	-	+	+	+	-	+	+	+	-	+	+	-
	OFC >2SDS	+	-	+	-	+	+	-	-	+	+	+	-	+	+	-
**Development**	Intell disab/glob dev delay	+	+	+	+	+	+	+	+	+	+	+	+	+	+	+
	Autistic/autistifom behavior	-	+	-	+	-	-	+	-	?	+	-	-	+	?	+
	Motor retardation	+	+	+	+	+	?	+	+	+	-	+	+	+	-	+
	Hypotonia	+	+	+	-	-	-	-	-	?	+	-	+	+	?	+
	Other behavioral anomalies	-	-	+	+	+	-	+	+	+	?	-	-	+	+	-
**Craniofacial**	Long /triangular face	+	+	+	+	+	+	+	+	+	+	+	-	+	+	+
	Prominent forehead	+	+	+	+	+	+	-	+	+	+	+	+	+	+	+
	Down-slanting palpebral fissures	+	+	+	+	-	+	-	+	+	+	+	+	-	+	+
	Deeply set eyes	+	-	+	+	-	+	+	-	-	+	-	-	+	+	+
	Depressed nasal bridge	-	-	-	-	-	-	-	-	-	-	-	-	-	-	-
	Short nose	-	-	-	-	-	-	-	+	-	-	+	-	-	-	-
	Upturned nasal tip/anteverted nares	-	-	+	-	+	-	-	-	+	+	-	-	-	-	-
	Long philtrum	-	-	-	-	-	-	-	+	-	-	+	-	-	-	+
	Small mouth	+	+	+	+	+	+	-	+	+	-	-	-	-	+	+
	Thin upper vermillion/ cupid bow	-	-	-	-	+	-	+	-	-	+	+	+	+	+	+
	Everted lower lip (E) /open mouth appearance (O)	E	-	E/O	E	-	E/O	E/O	E/O	E/O	E/O	E/O	E	E/O	E	O
	Prominent chin	+	-	+	+	+	+	-	+	+	+	+	-	+	+	-
**Eyes**	Vision impaired:H/S/N/M/A/O	S,M	S,N	S	S	-	^	^	H	S	-	H,S	N	S	-	H,O
	Blue sclerae	+	-	-	-	-	-	+	+	-	-	-	-	-	-	-
**Skeletal**	Slender habitus	+	+	-	+	+	-	-	+	+	+	-	-	+	+	+
	Kyphoscoliosis	+	-	-	-	+	-	-	-	-	+	-	-	-	-	-
	Pectus excavatum/carinatum	+	-	-	+	+	-	-	-	+	-	-	-	-	-	-
	Long hands	+	+	-	+	+	?	-	?	?	?	-	-	+	-	+
	Advanced bone age	+	?	-	-	+	?	+	+	-	-	?	?	+	-	+
	Joint laxity	+	-	+	-	-	-	-	+	?	?	-	-	-	-	-
**Brain MRI**	Nl/WV/C/BA/CM	CM	?	CM,WV	-	-	-	WV,*	-	-	?	?	-	?	-	-
**Seizures/EEG anomalies**		-	-	-	-	-	+	-	-	-	+	-	-	-	-	-

ID		16	17	18	19	20	21	22	23	24	25	26	27	28	29	30
**Gender**		M	F	F	M	M	F	M	M	M	F	F	M	M	F	F
**Age**		14y	10y	17y	24y	4y	2yr	4y	7yr	5y	6y	8y	42y	15y	12y	6y
**Prenatal growth**	Weight>2SDS	-	-	-	-	-	-	-	-	-	-	-	-	+	-	-
	Length>2SDS	-	?	-	-	-	-	-	?	?	?	-	-	+	-	-
	OFC >2SDS	?	?	?	-	-	-	-	?	-	?	+	+	-	-	-
**Postnatal growth**	Height >2SDS	+	-	?	+	+	-	-	-	-	-	+	+	+	-	+
	OFC >2SDS	+	+	+	+	+	+	-	+	+	+	+	+	+	+	+
**Development**	Intell disab/glob dev delay	+	+	+	+	+	+	+	+	+	+	+	+	+	+	+
	Autistic/autistifom behavior	?	-	+	+	-	-	-	-	-	-	+	+	-	-	-
	Motor retardation	+	?	+	+	+	+	+	+	?	+	+	+	-	+	+
	Hypotonia	?	-	+	+	+	+	+	-	-	+	+	+	+	+	+
	Other behavioral anomalies	+	-	+	+	-	-	-	+	-	+	+	+	+	+	+
**Craniofacial**	Long /triangular face	+	+	+	+	-	+	+	+	+	-	+	+	+	+	+
	Prominent forehead	+	+	+	+	+	+	+	+	+	+	+	+	+	+	+
	Down-slanting palpebral fissures	+	-	-	+	+	-	+	+	+	-	+	+	+	+	+
	Deeply set eyes	-	+	+	-	+	-	-	+	+	+	+	+	+	+	-
	Depressed nasal bridge	-	+	-	-	+	+	-	+	+	+	-	-	-	-	+
	Short nose	+	+	-	+	+	-	-	-	+	+	+	-	-	+	-
	Upturned nasal tip/anteverted nares	+	+	+	-	+	+	+	+	-	+	-	-	-	-	+
	Long philtrum	+	-	-	-	+	+	-	-	+	+	+	-	-	+	+
	Small mouth	+	-	+	+	+	-	+	-	+	+	+	+	-	+	-
	Thin upper vermillion/ cupid bow	+	+	+	-	+	+	-	+	+	+	+	+	-	+	-
	Everted lower lip (E) /open mouth appearance (O)	-	-	E/O	-	E	E/O	E	E/O	E/O	-	E/O	-	E/O	E/O	-
	Prominent chin	+	+	+	+	+	+	-	+	+	-	+	+	-	+	+
**Eyes**	Vision impaired:H/S/N/M/A/O	-	-	O	A	S,A	S,N,O	-	M,^	^	-	S,M,^	M	S	S,N,^	H §
	Blue sclerae	+	-	-	-	+	-	-	+	-	-	-	-	-	-	-
**Skeletal**	Slender habitus	+	+	-	-	-	+	-	+	+	-	-	+	+	+	+
	Kyphoscoliosis	-	-	-	-	-	-	-	-	-	-	-	-	+	-	+
	Pectus excavatum/carinatum	-	-	-	-	-	-	-	-	-	-	-	-	-	+	-
	Long hands	+	?	-	+	+	-	-	+	?	-	+	+	+	+	?
	Advanced bone age	-	?	+	+	?	?	?	+	+	?	+	+	+	-	+
	Joint laxity	+	-	-	-	-	-	-	-	?	-	+	-	-	+	?
**Brain MRI**	Nl/WV/C/BA/CM	-	-	-	-	WV,C	C,BA	?	+°	?	?	-	?	CM	WV	C
**Seizures/EEG anomalies**		+	-	+	-	-	-	-	-	-	-	-	-	-	+	-

ID		31	32	33	34	35	36	37	38	39	40	41	42	TOTAL
**Gender**		M	F	M	M	F	M	M	M	M	M	M	M	16F/26M
**Age**		11y	12y	16y	13y	3y8m	4y2m	22y	21y	3y	3y	10y	21y	
**Prenatal growth**	Weight>2SDS	+	-	?	-	-	-	-	-	-	-	-	-	4/40
	Length>2SDS	?	-	?	-	-	-	+	-	-	-	?	-	5/28
	OFC >2SDS	?	-	?	+	-	+	+	-	+	?	?	+	12/25
**Postnatal growth**	Height >2SDS	+	-	+	-	-	-	+	-	-	-	+	-	21/41
	OFC >2SDS	+	+	+	+	+	+	+	+	+	-	+	+	34/42
**Development**	Intelll disab/glob dev delay	+	+	+	+	+	+	+	+	+	+	+	+	42/42
	Autistic/autistifom behavior	+	-	-	-	-	+	+	+	-	+	+	-	16/39
	Motor retardation	+	-	+	?	+	+	+	?	+	+	+	+	32/37
	Hypotonia	-	-	+	-	+	+	+	?	+	+	-	+	25/38
	Other behavioral anomalies	+	-	+	+	-	+	+	+	-	+	+	-	26/41
**Craniofacial**	Long /triangular face	+	-	+	-	+	+	+	+	-	-	+	+	35/42
	Prominent forehead	+	+	-	+	+	+	+	+	+	+	+	+	40/42
	Down-slanting palpebral fissures	-	+	+	-	+	+	+	-	-	-	-	-	28/42
	Deeply set eyes	+	-	-	-	+	+	+	+	+	+	+	+	28/42
	Depressed nasal bridge	+	-	-	-	+	-	-	-	+	-	+	+	12/42
	Short nose	+	+	-	-	-	+	-	+	+	-	-	+	17/42
	Upturned nasal tip/anteverted nares	-	+	+	+	-	+	-	+	+	-	-	-	19/42
	Long philtrum	-	-	-	-	-	-	-	+	+	-	-	-	13/42
	Small mouth	-	-	+	-	+	+	+	+	+	-	-	-	26/42
	Thin upper vermillion/ cupid bow	-	+	+	+	+	+	+	-	+	-	-	-	26/42
	Everted lower lip (E) /open mouth appearance (O)	E/O	E/O	E/O	E/O	E/O	O	O	E/O	E/O	E/O	E	O	E:30/42<br>O:28/42
	Prominent chin	-	-	+	+	+	+	+	-	+	-	-	+	30/42
**Eyes**	Vision impaired:H/S/N/M/A/O	-	M	S	-	S,^	-	H,S,N,MA ,O	A,S,ç	S	S,O^	O^	^	32/42
	Blue sclerae	-	-	-	+	+	-	-	-	+	-	-	-	9/42
**Skeletal**	Slender habitus	+	-	+	-	+	+	+	+	-	-	+	-	25/42
	Kyphoscoliosis	+	+	+	+	-	+	+	+	-	-	-	-	12/42
	Pectus excavatum/carinatum	+	-	+	-	+	-	+	+	+	-	+	-	12/42
	Long hands	+	-	+	-	+	-	+	-	-	+	+	-	19/35
	Advanced bone age	+	-	?	+	?	?	+	+	+	?	?	?	19/27
	Joint laxity	+	-	-	-	+	-	-	+	+	+	+	-	12/38
**Brain MRI**	Nl/WV/C/BA/CM	?	-	-	?	WV	-	C	-	-	-	WV, C	WV	13/33<br>7WV 5C 3CM
**Seizures/EEG anomalies**		-	+	-	-	-	-	+	-	-	-	+	-	8/42

?unknown; H: Hypermetropia; S:Strabismus; N: Nystagmus

s; M: myopia; A: Astigmatism; O: optic disk pallor; WV: Wide ventricles; C: Corpus callosum underdevelopment; BA:Brain atrophy;

CM: Chiari malformation

^ Underdeveloped optic nerve

§ left papillar coloboma

* Mild periventricular leukomalacia, enlarged extra-axial space frontally, bilateral periventricular nodular heterotopia (R>L), subtle cortical dysplasia

° Reduced volume white matter posteriorly, small area heterotopia at posterior lateral ventricle

Ç tunnel vision

Table Ib. Patients with Malan Syndrome and Variants in *NFIX* Reported in Literature.

		Malan 2010	Yoneda 2012		Priolo 2012	Klaassen 2015		Gurrieri 2015			Martinez 2015		Jezela-Stanek 2016	Oshima 2017	Lu 2017	Total
**Gender**		F	F	M	F	F	M	F	F	M	M	F	F	M	M	6M/8F
**NFIX mutation**		p.(Gln190*)	p.(Le60Pro)	p.(Arg121Pro)	p.(Glu53_glu59del)	p.(Trp30Cysfs*24)	p.(Gln338*)	p.(Arg116Pro)	p.(Lys125Glu)	p.(Lys64Serfs*30)	p.(Arg38Cys)	p.(Arg54Pro)	p.(Arg115Trp)	p.(Asp90Glufs*29)	p.(Lys125Asn)	
**Prenatal growth**	Weight>2SDS	-	-	-	+	-	-	-	-	-	-	-	-	-	-	1/14
	Length>2SDS	-	-	-	+	?	?	-	-	-	-	-	+	-	-	2/12
	OFC >2SDS	+	-	-	+	?	?	-	+	-	+	-	-	+	+	6/12
**Postnatal growth**	Height >2SDS	+	-	+	+	+	-	+	+	+	+	+	-	+	-	10/14
	OFC >2SDS	+	-	?	+	+	-	+	+	+	+	+	-	+	+	10/13
**Development**	Intellectual disability/glob dev delay	+	+	+	+	+	+	+	+	+	+	+	+	+	+	14/14
	Autistic/autistifom behavior	+	-	-	+	-	-	-	-	-	-	-	?	-	-	2/12
	Motor retardation	-	+	+	+	+	+	+	+	+	+	+	+	?	+	12/13
	Hypotonia	-	+	+	+	+	-	+	+	+	+	+	+	?	+	11/13
	Other behavioral anomalies	+	?	-	+	-	-	+	+	+	+	+	+	-	-	8/13
**Craniofacial signs**	Long /triangular face	+	+	+	+	+	-	+	+	+	+	+	+	+	?	12/13
	Prominent forehead	+	+	+	+	+	+	+	+	+	+	+	+	+	?	13/13
	Down-slanting palpebral fissures	+	+	-	+	+	+	+	+	+	+	-	+	+	?	11/13
	Deeply set eyes	+	-	-	+	-	-	-	-	-	+	-	-	?	?	3/12
	Depressed nasal bridge	-	+	+	+	-	+	+	+	+	-	-	-	?	?	7/12
	Short nose	-	+	-	+	+	+	+	+	+	+	+	-	?	?	9/12
	Upturned nasal tip/anteverted nares	-	+	-	+	+	+	+	+	+	+	+	-	?	?	9/12
	Long philtrum	-	+	+	+	+	-	+	+	+	+	+	-	?	?	12/12
	Small mouth	+	+	-	+	+	+	+	+	+	+	+	-	?	?	10/12
	Thin upper vermillion/cupid bow	-	+	-	+	+	+	+	+	+	+	-	+	?	?	9/12
	Everted lower lip (E) /open mouth appearance (O)	E,O	E,O	O	E,O	E.O	E,O	E,O	E,O	E,O	E,O	O	E,O	?	?	E:10/12 O:12/12
	Prominent chin	+	+	+	+	+	-	+	+	+	-	+	+	+	?	11/13
**Eyes**	Vision impaired:H/S/N/M/A/O	S,N	S	-	S,N, O	S	S	-	O	O	S	S	S	-	-	10/14
	Blue sclerae	-	?	-	+	?	?	+	+	+	+	-	+	-	?	6/10
**Skeletal**	Slender habitus	+	+	+	+	-	-	+	+	+	+	+	+	-	?	10/13
	Kyphoscoliosis	+	+	-	+	-	-	-	-	-	-	+	-	+	-	5/14
	Pectus ecavatum/carinatum	-	+	-	+	+	-	+	+	+	-	-	-	+	-	7/14
	Long hands	-	+	?	+	-	-	+	+	+	+	+	+	+	?	9/12
	Advanced bone age	+	+	?	+	+	+	+	+	+	+	-	?	+	?	10/11
	Joint laxity	+	?	?	+	-	-	+	+	+	+	-	+	+	?	8/11
**Brain MRI**	Nl/WV/C/BA/CM	WV	?	?	C, ^	WV, #	-	WV,C	WV,C	-	-	-	WV	?	-	6/11 5wv/3c
**Seizures/EEG Anomalies**		-	+	-	-	-	-	-	-	-	-	-	+	+	-	2/13

? unknown; H: Hypermetropia; S:Strabismus; N: Nystagmus; M: myopia; A: Astigmatism; O: optic disk pallor; WV: Wide ventricles; C: Corpus callosum underdevelopment; BA:Brain atrophy; CM: Chiari malformation; ^ Underdeveloped optic nerve ; # periventricular white matter hyperintensity, possible heterotopias and a persistent cavum septum pellucidum

Table II. Patients with Deletions of Complete *NFIX*in the Present Report and Reported in Literature.

		Present report			Auvin 2009	Malan 2010	Dolan 2010	Nimma-kayalu 2013	Natiq 2014	Shimo-jima 2014	Klaassens 2015	Jorge 2015	Jazela-Stanek 2016	Dong 2016	Kuroda 2017	Total
		43	44	45												
		M	M	F	M	1M/1F	3F/1M	2F	F	3F	2M/2F	F	F	M	F	8M/16 F
**Age**		9y	17y	21y												
**Prenatal growth**	Birth weight (g)>2SDS	-	?	-	-	1/2	3/4	?	-	0/3	1/4	-	-	+	-	6/21
	Birth height (cm)>2SDS	-	?	-	-	1/2	?	?	-	0/3	0/4	-	+	-	-	2/17
	OFC (cm)>2SDS	+	?	+	-	1/2	3/3	?	-	1/3	0/4	-	-	-	-	7/21
**Postnatal growth**	Height >2SDS	-	+	-	+	2/2	3/4	1/2	+	1/3	2/4	+	-	-	-	13/24
	OFC >2SDS	+	+	-	+	2/2	4/4	2/2	+	1/3	1/4	+	+	-	-	16/24
**Development**	Intellectual disability/glob dev dleay	+	+	+	+	2/2	4/4	2/2	+	3/3	4/4	+	+	+	+	24/24
	Autistic/autistifom behavior	+	+	-	-	2/2	0/4	0/2	-	0/3	1/4	?	?	-	-	5/22
	Motor retardation	+	+	+	+	1/2	4/4	2/2	+	3/3	4/4	+	+	+	+	23/24
	Hypotonia	+	+	+	+	2/2	3/4	2/2	+	3/3	2/4	+	+	+	+	21/24
	Other behavioral anomalies	+	-	+	-	2/2	?	0/2	-	0/3	1/4	?	+	?	-	6/21
**Craniofacial**	Long (L) /triangular (T) face	+	+	+	+	2/2	4/4	2/2	+	3/3	2/4	+	-	+	-	20/24
	Prominent forehead	+	+	+	+	2/2	4/4	2/2	+	3/3	4/4	+	+	+	+	24/24
	Down-slanting palpebral fissures	+	+	-	?	1/2	1/4		+	2/3	3/4	?	-	?	-	11/21
	Deep set eyes	+	+	+	+	1/2	2/4	0/2	+	2/3	1/2	?	-	+	+	13/21
	Depressed nasal bridge	+	-	-	-	1/2	1/4	0/2	-	1/3	1/2	?	-	+	+	7/21
	Short nose	+	-	-	+	2/2	3/4	0/2	+	2/3	2/2	?	-	+	+	14/21
	Upturned Nasal tip/anteverted nares	+	-	-	+	2/2	1/4	0/2	+	3/3	2/2	+	+	+	+	15/22
	Long philtrum	+	-	+	+	0/2	2/4	1/2	+	2/3	2/2	+	-	-	+	13/22
	Small mouth	-	-	-	+	1/2	1/4	1/2	-	2/3	2/2	?	+	+	-	10/21
	Thin upper vermillion/cupid bow	+	-	+	+	1/2	2/4	1/2	+	3/3	2/2	?	+	+	+	16/21
	Everted lower lip (E) /open mouth appearance (O)	E,O	E,O	O	E,O	2/2 E,O	4/4	0/2	E,O	1/3	2/2 E,O	?	E,O	E,O	O	E:14/21 O:16/21
	Pointed/prominent chin	+	+	-	+	2/2	3/4	2/2	+	1/3	2/2	?	-	+	+	16/21
**Eyes**	Vision impaired:H/S/N/M/A/O	S,N,M,O	-	S	-	2/2H,S/ H,A	4/4 (*)	2/2S/S	S	0/3	3/4 (°)	S,N	S	S	S	18/24
	Blue sclerae	+	-	-	?	1/2	2/4	?	-	?	?	?	-	-	?	4/13
**Skeletal**	Slender habitus	+	+	+	-	2/2	1/4	1/2	-	2/3	0/4	+	+	-	?	11/23
	Kyphoscoliosis	+	+	+	-	1/2	?	0/2	-	?	1/4	?	-	-	-	5/16
	Pectus excavatum/carinatum	+	+	+	-	2/2	?	1/2	-	?	3/4	+	-	+	-	11/19
	Large long hands	+	+	+	-	2/2	2/2	1/2	-	?	1/1	?	+	-	-	10/15
	Advanced bone age	+	+	-	+	2/2	1/1	?	+	?	1/1	+	+	?	+	11/12
	Joint laxity	+	+	-	-	2/2	?	1/2	-	?	?	?	+	-	-	6/12
**Brain MRI**	Nl/WV/C/BA/CM	WV,C	?	-	-	1/2 WV,C	3/4(*)	1/2 C	BA	2/3 (&)	?	-	-	C	WV	11/19 4WV/6c/2cm
**Seizures/EEG anomalies**		+	-	-	+	0/2	2/4	1/2	+	2/3	1/4	-	+	-	+	11/24

?unknown; H: Hypermetropia; S:Strabismus; N: Nystagmus; M: myopia; A: Astigmatism; O: optic disk pallor WV:Wide ventricles; C: corpus callosum underdevelopment; BA:brain atrophy; CM: Chiari malformation; (*) In Dolan series. Vision impaired pz1: S,N; pz2: S,O and optic nerve underdevelopment; pz3: S, O, pz4: S,N,O and optic nerve

underdevelopment; Brain MRI Pz2: mild atrophy of frontal lobes; pz3: C, cystic lesion; pz4: WV; (°) In Klaassens series. Pz1: S,N; Pz2: S; Pz3: S; (&) In Shimoima series. Pz1 CM type1; Pz2: Cm type1, mildly reduced volume of the white matter, Pz3 Cm type1

Fig. S8. Clinical phenotype of presently reported 42 individuals with Malan syndrome.

Numbers of individuals in the panels correspond to numbers in the Tables. Ages are mentioned below each picture. For detailed descriptions please see Tables and text.

A. Side facial view of a limited number of presently reported individuals.

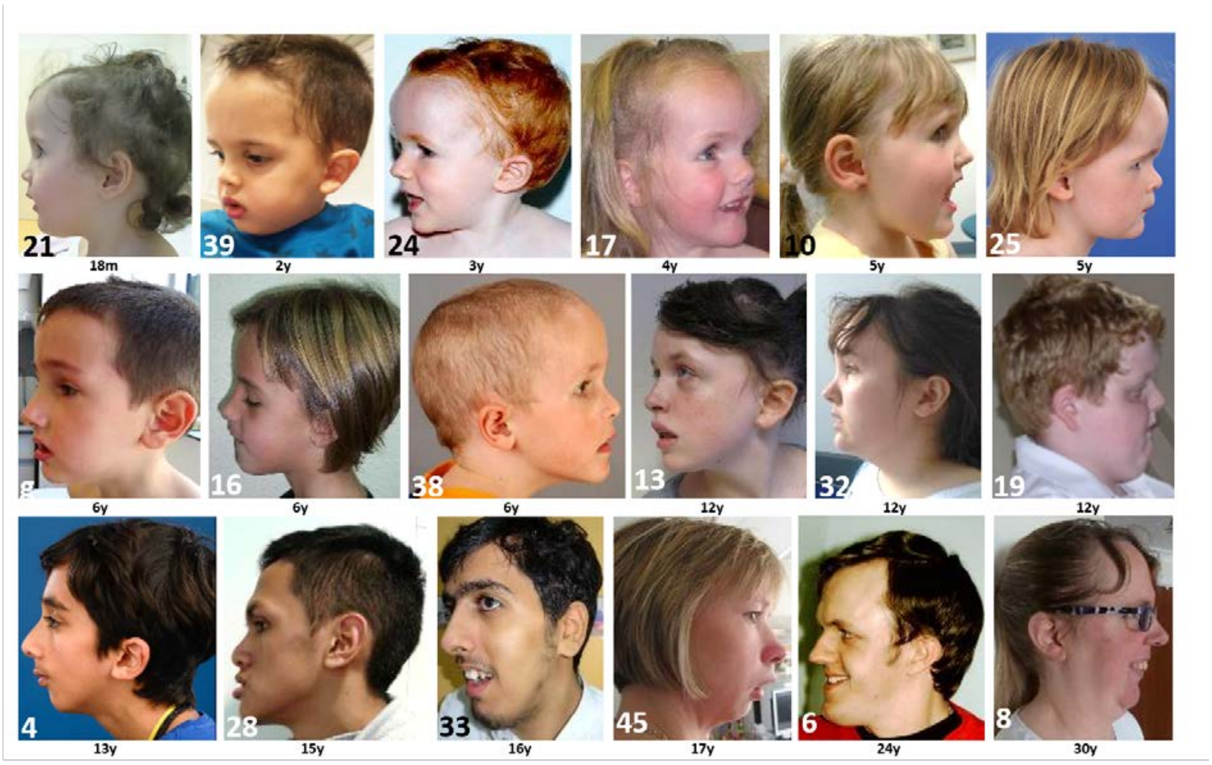

B. Distal limb characteristics and general body build in a limited number of presently reported individuals.

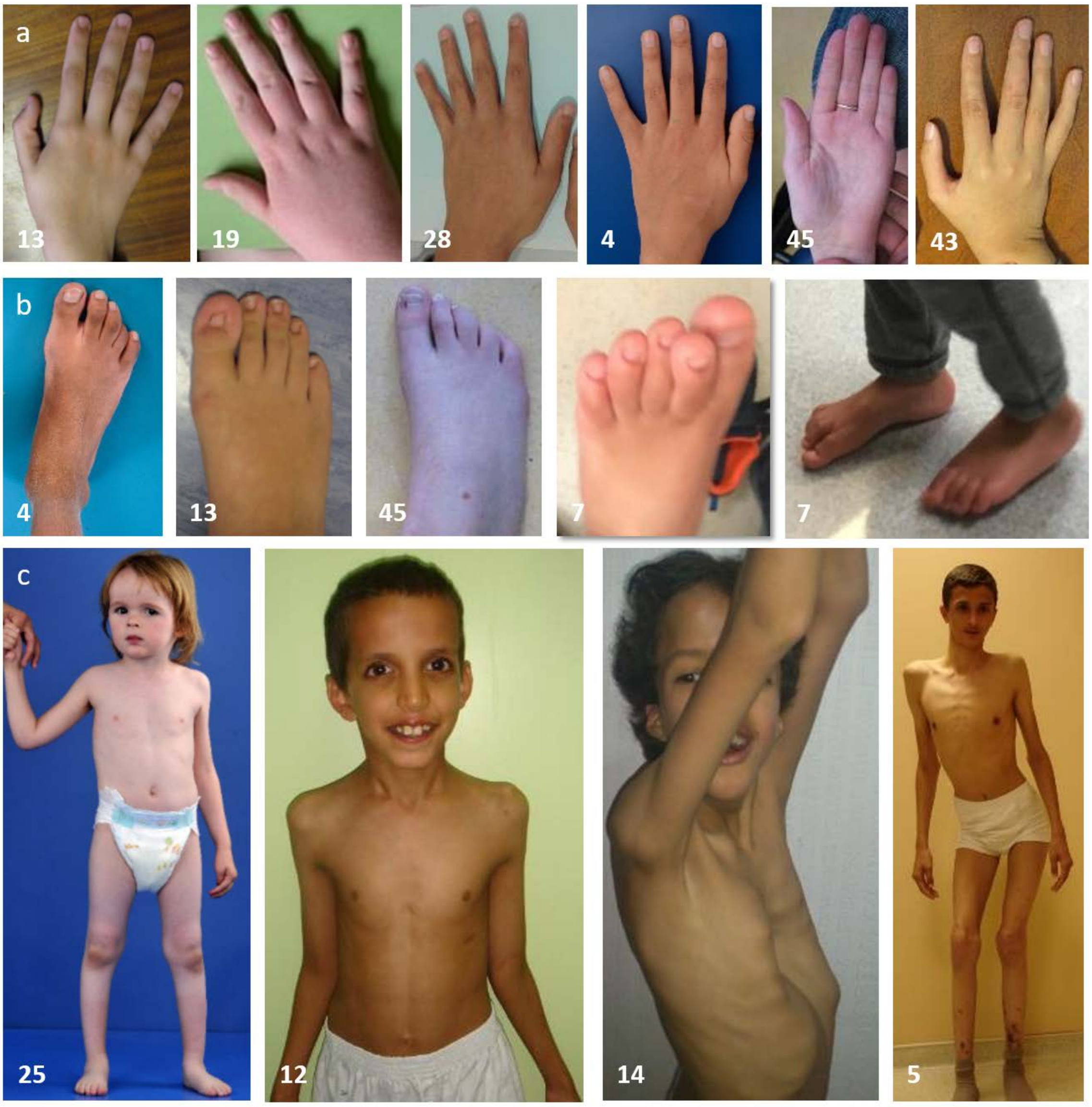

C. Changes with age in a limited number of presently reported individuals.

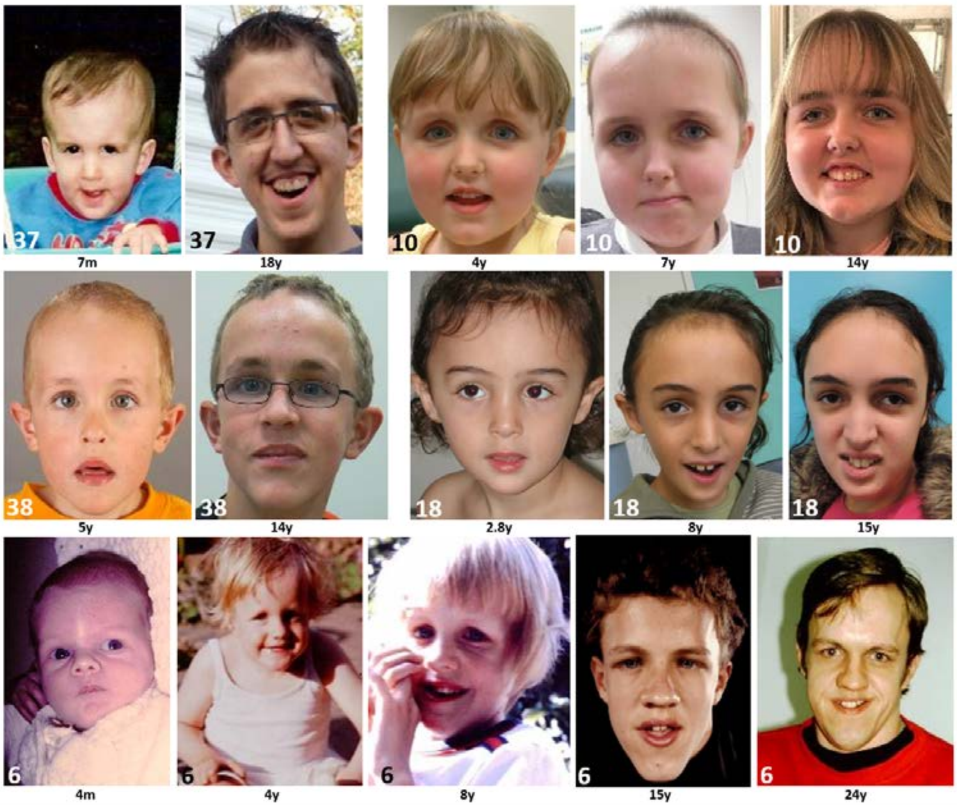

Supplement: Supplementary file 1 — Supporting information [file HUMU-39-1226-s001.pdf]
